# Programmed genome rearrangements in *Oxytricha* produce transcriptionally active extrachromosomal circular DNA

**DOI:** 10.1093/nar/gkz725

**Published:** 2019-08-28

**Authors:** V Talya Yerlici, Michael W Lu, Carla R Hoge, Richard V Miller, Rafik Neme, Jaspreet S Khurana, John R Bracht, Laura F Landweber

**Affiliations:** 1 Department of Biochemistry and Molecular Biophysics, Columbia University, New York, NY 10032, USA; 2 Department of Molecular Biology, Princeton University, Princeton, NJ 08544, USA; 3 Department of Biological Sciences, Columbia University, New York, NY 10027, USA; 4 Department of Biology, American University, Washington, DC 20016, USA

## Abstract

Extrachromosomal circular DNA (eccDNA) is both a driver of eukaryotic genome instability and a product of programmed genome rearrangements, but its extent had not been surveyed in *Oxytricha*, a ciliate with elaborate DNA elimination and translocation during development. Here, we captured rearrangement-specific circular DNA molecules across the genome to gain insight into its processes of programmed genome rearrangement. We recovered thousands of circularly excised Tc1/*mariner*-type transposable elements and high confidence non-repetitive germline-limited loci. We verified their *bona fide* circular topology using circular DNA deep-sequencing, 2D gel electrophoresis and inverse polymerase chain reaction. In contrast to the precise circular excision of transposable elements, we report widespread heterogeneity in the circular excision of non-repetitive germline-limited loci. We also demonstrate that circular DNAs are transcribed in *Oxytricha*, producing rearrangement-specific long non-coding RNAs. The programmed formation of thousands of eccDNA molecules makes *Oxytricha* a model system for studying nucleic acid topology. It also suggests involvement of eccDNA in programmed genome rearrangement.

## INTRODUCTION

**Figure 1.**
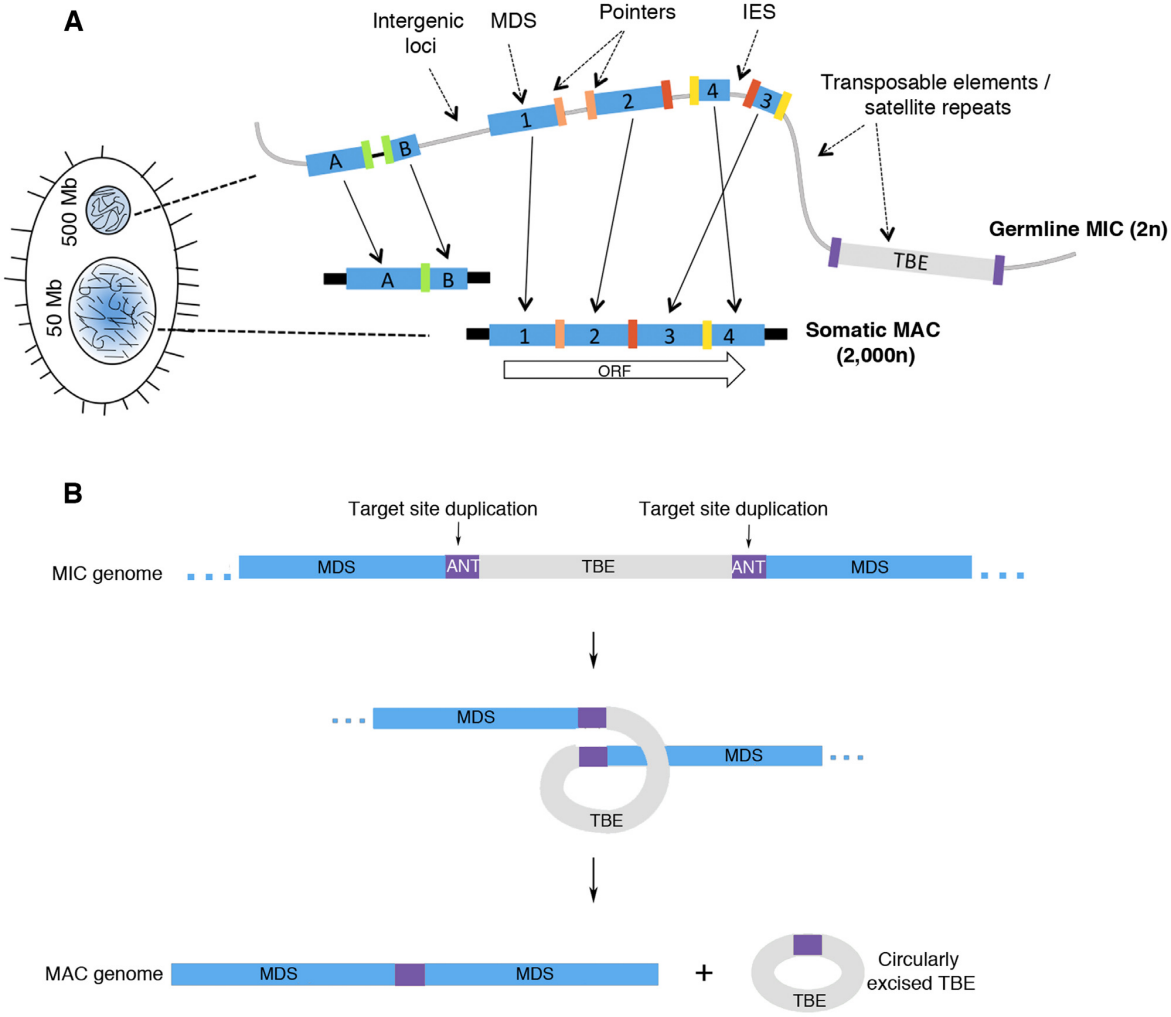
Genome rearrangements in *Oxytricha*. (**A**) Cartoon illustrating *Oxytricha*’s dual genome architectures. The germline micronuclear genome comprises of long chromosomes that mostly contain germline-limited sequences (gray line). Somatic macronuclear nanochromosomes contain only MDSs (blue rectangles). MDSs belonging to two different MAC nanochromosomes are labeled with letters or numbers. Short colored blocks with matching color denote *pointers*, short direct repeats that are present downstream of the *n*th MDS and upstream of (*n* + 1)th MDS. Black blocks at the ends of nanochromosomes denote telomeres, added *de novo* during genome rearrangement. The chromosome copy numbers are represented by 2n and ∼2000n for the MIC and MAC genomes, respectively. (**B**) A model for circular TBE elimination during rearrangement. Elimination of transposon-like sequences called TBEs (in gray) via precise excisions at the ANT target site duplication (purple) and subsequent circularization based on previous work is shown (17).

Ciliates are unicellular eukaryotes that undergo an exaggerated form of genome-wide DNA rearrangement and nuclear differentiation as part of post-zygotic development. Despite being unicellular, ciliates harbor two types of genomes in respectively different nuclei: a transcriptionally silent 500 Mb germline micronucleus (MIC) (1) and a transcriptionally active 50 Mb somatic macronucleus (MAC) that derives from a copy of the germline (2). The MIC is made up of ∼120 megabase length chromosomes (3), while the MAC contains over 16 000 nanochromosomes that are on average just 3.2kb long (2) (Figure 1A). Following meiosis, a cascade of programmed genome rearrangement events transforms the zygotic MIC into a new MAC. This process includes fragmentation of long MIC chromosomes, removal of MIC-specific sequences, splicing of genic MAC sequences and *de novo* addition of telomeres to mature MAC chromosomes (Figure 1A). MIC-specific sequences constitute 90–95% of the MIC genome and include repetitive sequences such as satellite repeats and transposable elements, as well as non-repetitive, mainly non-coding DNA segments, referred to as ‘internally eliminated sequences’ (IESs) that interrupt genic segments or ‘macronuclear-destined sequences’ (MDSs) (3–5) (Figure 1A).

In *Oxytricha* and a few other ciliate lineages (6–8), MDSs may be present in a scrambled, or non-linear, order or orientation on the germline MIC chromosomes and programmed genome rearrangement must precisely join them in the correct, linear, order, especially since most MDS junctions lie within ORFs (1,9,10). Two ncRNA pathways are known to participate in MDS assembly in *Oxytricha*. Rearrangement-specific maternal, long *template RNAs*, transcribed from full-length MAC chromosomes, guide MDS unscrambling (11,12), while 27 bp piRNAs mark MDSs for retention in the new MAC (13,14). Furthermore, short direct repeats, called *pointers*, are present at the boundaries of MDSs that are consecutive in the MAC, and these are thought to facilitate rearrangement (1), with their alignment guided by lncRNA templates (11).

A limited number of cases of extrachromosomal circular DNA (eccDNA) formation during genome rearrangements have been studied in the ciliates *Euplotes* (15,16), *Oxytricha* (17) and *Paramecium* (18). Furthermore, as drivers of genome plasticity, eccDNAs are involved in myriad biological phenomena. In yeast, eccDNAs are involved in gene amplification, facilitating adaption to nutrient-limiting environments (19,20) as well as senescence caused by rDNA circles (21). Similarly, in plants, eccDNA has a role in transmissible herbicide resistance (22), while in human tumor cells eccDNA may drive increases in oncogene copy number (23,24). In addition to influencing gene expression through DNA copy number variation, circular DNAs arise during immunoglobulin class switch recombination during development of vertebrate adaptive immunity (25). eccDNAs also originate from repetitive loci such as satellite repeats (26–28) and are important intermediates for transposable element mobility (29). Moreover, recent findings suggest possible roles of eccDNA transcription in both the down-regulation of miRNA-mediated expression in humans (30), as well as small RNA regulation of DNA deletion in *Paramecium* (31).

Previous work demonstrated that transposon-like sequences called telomere-bearing elements (TBEs) in *Oxytricha* are excised as eccDNA molecules during rearrangement (17) (Figure 1B). *Oxytricha* harbors an estimated 34 721 partial or complete copies of these Tc1/*mariner* transposons in its MIC, which collectively constitute ∼13% of the MIC genome (32). An important feature of TBEs is the presence of terminal inverted repeats (TIRs), which contain terminal telomeric sequences (G_4_T_4_)_2_G (33). TBEs also exhibit an ANT flanking target site duplication (TSD), which is thought to be the preferred site for integration, similar to the AT TSD of Tc1/*mariner* elements in other organisms (34).

It is unknown whether non-repetitive MIC-limited sequences are also removed as eccDNA during genome rearrangement in *Oxytricha*, and whether these are simple DNA elimination byproducts. Furthermore, the mechanism of DNA breakage and repair that leads to removal of TBEs and other non-repetitive MIC-limited sequences during programmed genome rearrangement is largely unknown. Here, we characterize eccDNA genome-wide during DNA rearrangement in *Oxytricha*. Using a high-throughput sequencing approach targeting eccDNA (Circulome-seq) (20,35,36), we capture circularized TBE sequences genome-wide during rearrangement. We find that eccDNA molecules from non-repetitive MIC-limited sequences are also abundantly produced during genome rearrangement. Circularization involves imprecise and heterogeneous cut sites in the vicinity of the pointer repeats that join consecutive MDSs. We also detect long, non-coding RNAs produced from eccDNAs and characterize variable and bidirectional transcription start sites (TSSs) in the vicinity of circle junctions, suggesting transcription of eccDNAs during genome rearrangement.

## MATERIALS AND METHODS

### *Oxytricha* culturing and mating

*Oxytricha trifallax* mating types JRB310 and JRB510 were maintained as described before (13), with the addition of 1:1000 *Klebsiella* grown overnight in LB broth (10 g/l tryptone, 10 g/l NaCl, 5 g/l yeast extract) (Sigma-Aldrich) every other day during asexual growth. Mating was induced by starving the cells overnight and mixing equal numbers of JRB310 and JRB510 at a 5000 cells/ml final concentration. Pairing was observed 2–3 h post-mixing with 70–95% maximum pairing efficiency by 12 h. Asexual cells were harvested immediately after mixing JRB310 and JRB510. Early, mid- and late rearrangement time points refer to 24, 36, 48 h post-mixing of cells of compatible mating types (Supplementary Figure S1) (37).

### DNA extraction and enrichment for circular DNA

About 1–2 million cells were harvested with the addition of 50 mM ethylenediaminetetraacetic acid (EDTA) to concentrated cell suspension and centrifuged for 1 min at 130 g. Asexual, early and mid-rearrangement samples were collected as two biological replicates. The cell pellets were flash frozen in liquid nitrogen and kept at −80°C until DNA extraction. Frozen cell pellets were lysed overnight at 55°C in 1× lysis buffer (100 mM NaCl, 10 mM Tris pH 8.0, 25 mM EDTA pH 8.0, 0.5% sodium dodecyl sulphate (SDS)) in a total volume of 450 μl with the addition of 0.5 μg/μl proteinase K (New England BioLabs). Whole-cell DNA was phenol-chloroform extracted from cell lysates and ethanol precipitated overnight at −20°C. All ethanol precipitations for DNA used as input for Nextera libraries were done with the addition of 0.002 volume linear acrylamide (Invitrogen). The DNA was resuspended in 100 μl nuclease-free water (Ambion). DNA was diluted to 50 ng/μl and RNA was removed with the addition of 10 ng/μl RNase A (Ambion) according to Shibata *et al.* (35), followed by phenol–chloroform extraction and ethanol precipitation. A total of 2 μg of whole-cell DNA was used in a 250 μl DNase reaction with 1× Plasmid-safe reaction buffer, 100U Plasmid-safe adenosine triphosphate (ATP)-dependent DNase (Lucigen) and 2mM ATP and incubated overnight at 37°C (20,35,36). Plasmid-safe ATP-dependent DNase was inactivated by incubating at 70°C for 30 min. DNase treatment was repeated with fresh ATP and DNase supplement for a total of three successive times. The circular DNA enriched fraction was then phenol-chloroform extracted and ethanol precipitated.

### Library preparation and sequencing

A total of 1 ng of eccDNA-enriched and unenriched sample was directly used as input for Nextera library preparation (Illumina) according to manufacturer's recommendations with 1–5 μl Amplicon Tagment Mix (ATM). We note than no polymerase chain reaction (PCR) was performed on the input DNA before the library preparation. After tagmentation we amplified the tagmented DNAs via 10 cycles of PCR. Libraries were sequenced on a MiSeq (paired-end 150 and 75 nt reads) at Princeton University.

### Circulome-seq read processing

Barcodes were split using Galaxy (38–40). Quality and Illumina adapter trimming was done using Trim Galore (Trim Galore version 0.4.3 http://www.bioinformatics.babraham.ac.uk/projects/trim_galore/, Cutadapt version 1.13 (41)) with the following parameters: –q 20 –e 0.1 –O 1. For comparison across the developmental time points, the 150 and 75 nt reads for the mid-rearrangement samples were pooled and trimmed to 75 nt length using Fastx_trimmer (http://hannonlab.cshl.edu/fastx_toolkit/). For normalization by sequencing depth, Bowtie2 –end-to-end with default parameters (42) was used to determine the sum of all MAC, TBE and other discordant or concordantly MIC mapping read pairs. This number was used to calculate RPM (reads per million mapped) and normalize for the variation in sequencing depths across the different libraries.

### Analysis of TBE junction reads

To find reads containing circular TBE junctions the regular expression ‘GGTTTTGGGGTTTT.A.T.AAAACCCCAAAACC’ was used, where ‘.’ denotes any single nucleotide. The number of reads containing circular TBE junctions was normalized for sequencing depth as described above and reported as RPMs.

### Finding eccDNA junction reads in non-repetitive MIC-limited loci

Paired reads were collapsed and handled like single-end reads in order to find junction-spanning reads in non-repetitive MIC-specific loci. Reads were mapped using Bowtie2 –end-to-end with default parameters to the MAC (2) and MIC assemblies (1) and fully mapping reads were removed. Unmapped and partially mapped reads were subsequently mapped to the MIC genome assembly again using BWA-MEM with default parameters (43) to find chimeric reads. Multi-mapping reads, reads with MAPQ < 5, PCR duplicates and secondary read flags were removed using SAMtools view –bSq 5 and view –F 1284 (44). At last, supplemental reads were extracted using samtools view –T –f 2048 and circular junctions identified using a custom Python script. Such chimeric reads mapping within 150 nt of the ends of MIC contigs were removed using BEDtools intersect (45) to filter out any assembly artifacts.

### Finding read pairs containing the signature 9 bp duplication

The 75 nt paired-end reads with matching 9 bp at the 5′ ends were identified and mapped to the MIC assembly using BWA mem with default parameters. SAM files were filtered to remove multi-mapping reads, reads with MAPQ < 5, PCR duplicates, secondary flags and supplementary flags. At last, read pairs where both pairs map in an orientation such that the 5′ ends overlap by exactly 9 bp were counted using a custom Python script. Reads mapping close to the ends of MIC contigs were removed as described above.

### Generating the genome tracks

The 75 nt trimmed reads were mapped to the MIC genome assembly using Bowtie2 –end-to-end with default parameters. SAM files were filtered as mentioned before. After filtering, the number of reads mapping from each library was used to subsample the reads using samtools view –s to normalize for different sequencing depths. To generate the genome tracks, BAM files were converted to bedgraph files using BEDtools genomecov and visualized using the R (version 3.4.1) package Sushi (Phanstiel, D.H. Sushi: tools for visualizing genomics data, version 1.14.0).

### Annotating and characterizing high confidence eccDNA specific to mid-rearrangement

Circle junction-spanning reads were used to determine putative eccDNA coordinates. Full-length 150 and 75 nt reads were mapped to the MIC genome assembly using BWA-MEM default parameters. Coverage within circle coordinates was determined using BEDtools coverage. To generate high confidence circle annotations, the list of putative eccDNA was filtered according to read coverage within these coordinates: (i) at least 25% coverage in the two +exo mid-rearrangement replicates where coverage denotes the fraction of the circle body covered with ≥1 read and (ii) ≤15% coverage in the two asexual samples. The distance between high confidence eccDNA and the nearest MDS boundary as well as direct repeats flanking the site of circularization were annotated using the custom Python scripts. The set of high confidence eccDNA annotations were randomized 500 times using BEDtools shuffle along the MIC assembly. For circles located at a distance of ±50 bp to MDS annotations, the circle start and end site with respect to direct repeats and what type of eliminated sequence they reside on was also determined using custom Python scripts. The histograms were generated using R (version 3.4.1).

### Two-dimensional agarose gel electrophoresis and Southern blotting

A total of 10 μg of whole-cell DNA was separated on a 0.4% SeaKem Gold agarose gel in 1× TAE without the addition of ethidium bromide (EtBr). The first dimension was run for 20 h at 20V at room temperature in 1× TAE. The lane was excised and rotated 90 degrees and embedded in 1% SeaKem Gold agarose with 0.6 μg/ml EtBr. This second dimension was run for 20 h at 44V at 4°C in 1× TAE containing 0.6 μg/ml EtBr. The gel was imaged on an AI600RGB to assess the quality of separation, then the DNA was depurinated and denatured before being transferred to an Amersham Hybond-N+ positively charged nylon membrane (GE Healthcare Life Sciences) through neutral capillary transfer with 20× saline sodium citrate buffer (SSC) for 24 h (46). After transferring, DNA was UV cross-linked to the membrane using an Ultra-Lum UVC-515 set to 70 000 micro-joules/cm2. Digoxigenin (DIG)-labeled probes were amplified from mid-rearrangement genomic DNA using the PCR DIG Probe Synthesis Kit (Roche) according to manufacturer's instructions, with the exception of using ¼ of the standard amount of DIG-labeled nucleotides. Probes were hybridized overnight using the DIG EasyHyb system (Roche) according to manufacturer's instructions. Chemiluminescent detection of hybridized probes was performed using anti-DIG-AP Fab fragments and CDP-Star substrate (Roche) and imaged on an Amersham AI600RGB. Membranes were stripped of hybridized probes using 0.2M NaOH containing 0.1% SDS according to manufacturer's instructions, then stored at 4°C in 2x SSC between hybridizations. Restriction digested DNA sample was cut using BglII (New England BioLabs) according to manufacturer's instructions, then phenol–chloroform extracted and ethanol precipitated before being separated on a 2D agarose gel. Supercoiled DNA ladder (New England BioLabs) and 1 Kb Plus DNA ladder (Invitrogen) were used as spike-in standards. The supercoiled DNA ladder was nicked using Nb.BtsI (New England BioLabs) to generate the relaxed circular DNA standard. Primers used for generating the probes are listed in Supplementary Table S1.

### DNA extraction for inverse PCR

Whole cell DNA was extracted from *Oxytricha* either by phenol-chloroform extraction and ethanol precipitation as described in ‘DNA extraction and enrichment for circular DNA’ or Nucleospin Tissue kit (Macherey-Nagel) according to manufacturer's instructions. Inverse PCR was performed using Phusion polymerase (New England BioLabs). The PCR amplicons were cloned using TOPO TA cloning kit (Invitrogen) and transformed into One Shot TOP10 chemically competent cells (Invitrogen). Clones were sequenced using M13F and M13R primers (Genewiz). Sanger sequencing traces were visualized using Geneious Pro 5.6.3 (https://www.geneious.com). Inverse PCR primers are listed in Supplementary Table S1.

### Terminal transferase tailing of 3′ DNA ends to map break points

Genomic DNA was tailed with dGTP using terminal transferase according to manufacturer's instructions (NEB). The G-tailed gDNA was amplified using 40 net cycles of nested PCR using either Qt, Qo and Qi primers or A(C) and A primers, in addition to gene-specific primers (Supplementary Table S1) and resolved on an agarose gel. Amplified products were gel extracted (Qiagen) and transformed into One Shot TOP10 chemically competent cells (Invitrogen). Colonies were screened for clones containing target fragment sizes consistent with possible cuts at MDS-IES boundaries. Isolated plasmids were Sanger sequenced (Genewiz) to map the precise 3′ DNA ends at MDS boundaries.

### qPCR

A total of 700 pg of pUC19 plasmid was spiked-in for 1 μg of whole-cell DNA to assess enrichment of circular DNA. Power SYBR Green PCR Master Mix (Applied Biosystems) and Biorad CFX384 Real-Time System were used for the qPCR assays to determine the relative levels of pUC19 circular spike-in, linear mitochondrial genome and TBEs in +exo and –exo samples. All qPCR reactions were done in technical triplicate or duplicate. A total of 1 ng of DNA before (–exo) and after (+exo) exonuclease treatment was used as input. Fold change was calculated using 2^(-ΔCt)^ where ΔCt = Ct_+exo_ – Ct_-exo_. TBE primers were the same ones used in generating the Southern probe. Additional qPCR primers are listed in Supplementary Table S1.

### RNA-seq library preparation, sequencing and data analysis

Approximately one quarter million cells at 12 and 36 h post-mating were harvested in triplicate. Total RNA was extracted using TRIzol Reagent (Invitrogen) and treated with Turbo DNase (Invitrogen) according to manufacturer's instructions. polyA+ RNA was isolated with the polyA mRNA isolation kit (NEB) according to manufacturer's instructions. Sequencing libraries were prepared using ScriptSeq (Epicentre) and sequenced on the Illumina HiSeq 2500 platform to obtain paired-end 75 nt reads. Reads were quality filtered using Trimmomatic (47) with options SLIDINGWINDOW:4:25, MINLEN:60, mapped to the MIC, MAC and transcriptome assemblies using BWA-MEM and processed with SAMtools and BEDtools. A random distribution of the transcriptional state was generated using 1000 random permutations of high confidence circle coordinates along the MIC assembly with BEDtools shuffle. Two types of permutations were performed, one to assess genome-wide transcription and another restricted to IES regions in the genome. Counts were normalized using an adjustment factor based on the ratio of mapped reads in each library to the library with the lowest count of mapped reads. The coverage of circles was obtained with BEDtools coverage. Normalization was performed by subsampling reads from each library, using an adjustment factor based on the ratio of mapped reads in each library to the library with the lowest count of mapped reads (SAMtools view –s).

### Reverse transcription coupled to inverse PCR

Total RNA was isolated as described above at 12 h intervals and reverse transcribed with SuperScript III (Invitrogen) using random hexamers according to manufacturer's instructions. cDNA was used as template for inverse PCR as described above.

### 5′-Rapid amplification of cDNA ends (5′-RACE)

5′-RACE was done as described in Scotto-Lavino *et al.* (48). Strand-specific, gene-specific primers were used to reverse transcribe 800 ng of DNase-treated total RNA using AMV reverse transcriptase according to manufacturer's instructions (NEB). cDNA was purified using MinElute (Qiagen) and 5 pmol of cDNA was terminal transferase-treated (NEB) to A-tail according to manufacturer's instructions. The A-tailed cDNA was amplified using 40 net cycles of nested PCR before resolving the products on an agarose gel. RACE products were gel extracted (Qiagen), transformed into One Shot TOP10 chemically competent cells (Invitrogen) and Sanger sequenced (Genewiz) to map the precise TSS in three validated eccDNAs. Primers Qt, Qo and Qi, which were also used in mapping 3′ DNA breaks, were used in combination with gene-specific primers (Supplementary Table S1).

## RESULTS

### Genome-wide sequencing reveals circularly excised Tc1/*mariner-*type telomere-bearing elements during genome rearrangement

**Figure 2.**
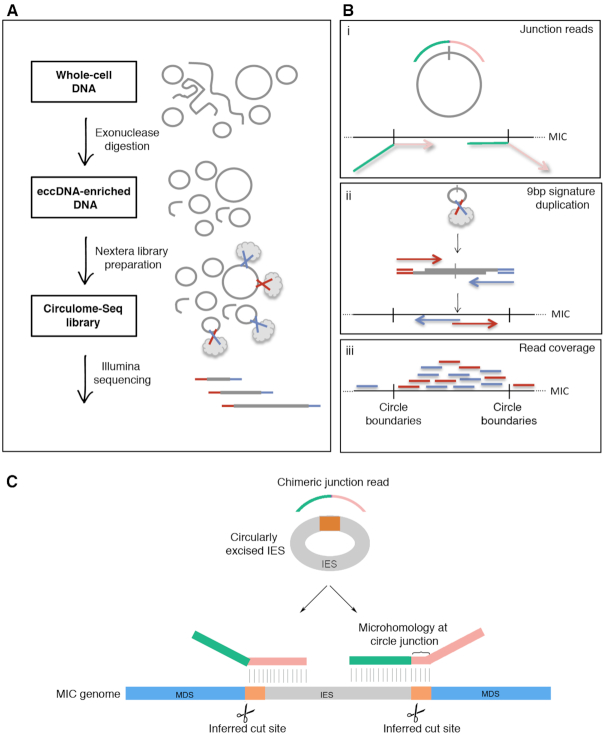
Experimental and bioinformatic pipeline to identify eccDNA in *Oxytricha* genome-wide. (**A**) Experimental pipeline to enrich for and sequence eccDNAs. Gray lines represent whole-cell DNA (either linear or circular). Gray clouds represent the Nextera transposome, which simultaneously fragments DNA and ligates the blue and red Illumina sequencing primers to the DNA fragments. (**B**) Three bioinformatic metrics are used to identify eccDNA: (i) Junction reads in which the 5′ and 3′ portions of chimeric-reads (green and pink) coming from circle junctions map to the linear MIC genome assembly in a permuted order, as shown, (ii) When a small circular DNA molecule is cut only once by the Nextera transposome, via a 9 bp staggered cut, it will produce a signature 9 bp duplication at the 5′ ends of the reads in a pair that is a bioinformatic signature for circular topology in non-repetitive regions, (iii) Exonuclease digested libraries will have increased coverage in loci that give rise to eccDNA, compared to non-exonuclease treated samples. Paired-end reads are represented as two lines where the two reads in a pair are red and blue. (**C**) Hypothetical pathway for the excision of an IES (in gray) via recombination at pointers (orange). The circular junction read is shown as part green and part pink to illustrate regions of partial MIC alignment, where the pink and green portions map upstream and downstream of the IES, respectively. The coordinates for the split alignments for a circular junction read can then be used to infer (i) cut sites that would lead to the excision of the IES and (ii) whether direct repeats flank the circularly excised loci.

Williams *et al.* previously described the circular excision of a Tc1/*mariner*-type TBE transposon during *Oxytricha* genome rearrangement (17) (Figure 1B). We used a sequencing-based approach, Circulome-seq (36), to interrogate eccDNA molecules genome-wide. Briefly, whole-cell DNA was purified both during asexual growth and at various time points during rearrangement, and then exonuclease digested to reduce the abundance of linear chromosomes (20,35). The eccDNA-enriched samples (+exo) as well as unenriched DNA samples (–exo) were used as input to prepare Nextera libraries that were sequenced on the Illumina platform (36) (Figure 2A). Two metrics were used to determine eccDNA counts: (i) chimeric reads that span the eccDNA junctions, referred to as junction reads (20,35) (Figure 2B and C) and (ii) 9 bp duplications that are created at the 5′ end of Illumina read pairs when a small eccDNA is cut and tagged once with the Nextera tagmentase (36) (Figure 2B).

**Figure 3.**
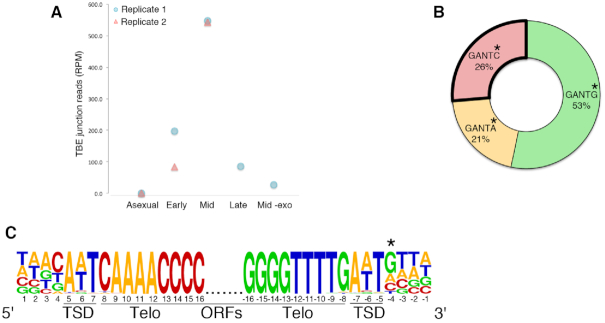
Circular DNA enrichment coupled to deep-sequencing reveals circularly excised TBEs. (**A**) Counts of Illumina reads that map to circular TBE junctions in the various samples across development were normalized to the total number of mapped reads in each library (RPM). The mid-rearrangement replicate 1 sample that was not treated with the exonuclease is represented by *–exo*. Asexual, early and mid-rearrangement samples were done as two biological replicates. (**B**) Circular DNA sequencing pipeline enriched for circularly excised TBE junction reads that can be classified into three groups according to the 5 bp central sequence at the junction. The outlined GANTC motif is the previously identified circular junction (17). (**C**) The consensus sequence (73) of the TIR from 2636 TBEs in the MIC genome assembly (1,32) was generated by searching for partially conserved A_2_C_4_A_4_C_4_ and G_4_T_4_G_4_T_2_ telomeric motifs at both ends, allowing for detection of variation internal to the TSD. Positions 5 to 7 and −7 to −5 are the ANT TSD. Positions 1 to 4 and −1 to −4 show the 4 nt flanking the TSD. The nucleotide distribution at position −4 (also marked with an asterisk) has the following nucleotide distribution: 19% C, 52% G, 22% A and 7% T. On the TBE circle junction motif, this nucleotide corresponds to the one marked with an asterisk in (B).

We first analyzed circulome-seq reads for the presence of circular TBEs during rearrangement, since circularly excised TBEs provide an internal control to validate eccDNA enrichment. Among the eccDNA enriched Illumina reads, circular TBE junction reads were exclusively present during rearrangement, with peak abundance during mid-rearrangement and absent during asexual growth (Figure 3A and Table 1). Moreover, TBE junction reads were enriched upon exonuclease treatment (+exo versus –exo mid-rearrangement samples; Figure 3A). Close examination of the TBE circle junction reads suggests that circular elimination of TBEs gives rise to three distinct classes of junctions according to the central 5 bp sequence at the ligation site: GANTC (17), GANTG and GANTA, where the central ANT is the TSD sequence (Figure 3B). To gain mechanistic insight into the cleavage of TBEs and understand the source of the different junction motifs, we examined the consensus sequence of TIRs and flanking nucleotides among 2636 TBEs in the MIC genome assembly (1,32) (Figure 3C). This analysis suggests that the nucleotides internal to the TSD (positions 8 and –8 in Figure 3C) are highly conserved and cannot account for the variability at the circular junction (Figure 3B). Intriguingly, the nucleotide immediately outside of the TSD (positions 4 and –4 in Figure 3C) has a non-random distribution (different from positions 1, 2, –1 and –2) (Figure 3C) that closely resembles the distribution of the circular junction motif (Figure 3B). This is suggestive of a 5 bp staggered cut, centered at the ANT TSD, during excision of TBEs.

**Table 1.** Circulome-seq read statistics

**Time point**	**Replicate ID**	**No. of mapped reads**	**No. of TBE junction reads**	**Normalized TBE junction read counts (RPM)**	**No. of circle junction reads**	**Unique circle isoform counts**	**Normalized non-repetitive circle junction read counts (RPM)**	**No. of 9bp duplication reads**	**Normalized 9bp duplication read counts (RPM)**
**Asexual**	1	1 271 072	0	0.0	33	27	26.0	3	2.4
	2	1 091 793	0	0.0	23	16	21.1	1	0.9
**Early**	1	993 575	197	198.3	74	71	74.5	5	5.0
	2	1 498 785	125	83.4	61	48	40.7	5	3.3
**Mid**	1	1 185 659	649	547.4	2176	2039	1835.3	176	148.4
	2	1 095 501	594	542.2	1464	1362	1336.4	72	65.7
**Late**	1	1 085 662	93	85.7	771	715	710.2	29	26.7
**Mid -exo**	1	1 543 270	42	27.2	199	182	128.9	73	47.3

To further confirm the circular conformation of TBEs during genome rearrangement, qPCR analysis indicated that exonuclease treatment does not significantly alter the levels of TBEs during mid-rearrangement, suggesting the presence of both circular and not-yet-excised linear elements, whereas during the asexual phase, TBEs (that are abundant in the MIC genome) are depleted upon exonuclease treatment (Supplementary Figure S2). qPCR also confirmed that exonuclease treatment leads to enrichment of a circular spike-in pUC19 plasmid and depletion of the linear mitochondrial DNA (49) at all time points, as expected (Supplementary Figure S2).

### Circularly excised non-repetitive MIC-limited loci are enriched during genome rearrangement

**Figure 4.**
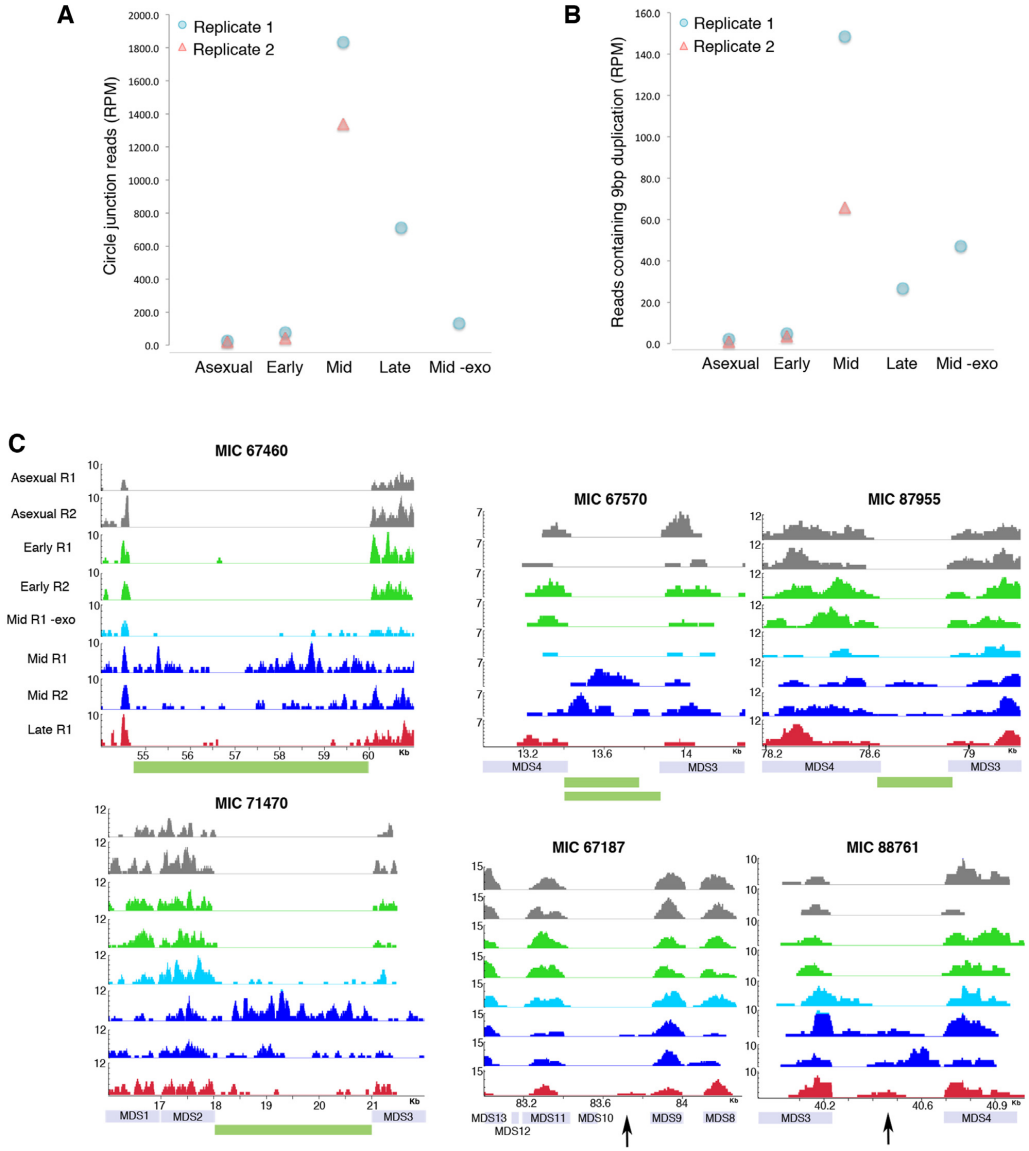
Circulome-seq also reveals circularly excised non-repetitive MIC-limited loci. (**A**) Circular junction reads in non-repetitive MIC-limited loci were counted and normalized to the total number of mapped reads in each library (RPM). (**B**) Paired-end reads containing the signature 9 bp duplication at the 5′ ends were counted and normalized to the total number of mapped reads in each library (RPM). (**C**) Example genome tracks during development showing mapped reads that were enriched for circular DNA as well as −exo that was not enriched (light blue track). Biological replicates are shown with the same color and are represented by R1 and R2. The *y*-axis represents sequencing coverage of reads that were subsampled to normalize for sequencing depth. Loci shown in MIC 67460, MIC 71470, MIC67570, MIC87955 were detected via circular junction reads and are annotated as high confidence circles (shown as green blocks). Loci shown in MIC 67187 and MIC 88761 contained paired-end reads with the 9 bp signature duplication shown with black arrows.

Having verified that our sequencing pipeline for eccDNA enriches for circularly excised TBEs, the Illumina reads obtained from these libraries were further analyzed to investigate the presence of other circular, non-repetitive MIC-limited sequences during development. While we observed low levels of junction reads during asexual growth, at a mid-point during rearrangement the junction read counts rose on average 67-fold (Figure 4A and Table 1). Furthermore, there was an increase in detectable junction reads in exonuclease-treated mid-rearrangement samples compared to –exo (Figure 4A and Table 1). Similarly, when read pairs containing the 9 bp signature duplication were counted, we found comparable changes in the read counts across the different libraries (Figure 4B and Table 1).

Using junction reads, we identified a total of 3896 and 2589 unique eccDNA sequences originating from non-repetitive MIC loci in the two mid-rearrangement samples, respectively, when eccDNA abundance is at its peak. We designate 2432 of these as high confidence, based on stringent filtering that takes into account read coverage in both +exo mid-rearrangement replicates, as well asexual replicates and the presence of at least one circle junction spanning read. This serves the purpose of eliminating eccDNA sequences that may be present in asexual growth and low copy number cases that may be hard to validate. In Figure 4C we show some examples of Circulome-seq data demonstrating the presence of eccDNA specifically in +exo mid-rearrangement samples, relative to asexual and –exo mid-rearrangement controls (Figure 4C). An important control for the exonuclease treatment is the linear mitochondrial genome (49), which was strongly reduced across all developmental time points, as measured by qPCR (Supplementary Figure S2). While it is possible that eccDNA is present in asexual cells, detection is complicated by the presence of linear MAC sequence that may be resistant to exonuclease treatment. Instead, we focus on high confidence eccDNA containing MIC-limited sequences. These are present exclusively during genome rearrangement, thus avoiding the artifactual detection of linear MAC.

### Genomic attributes of rearrangement-specific high confidence eccDNA

We detect high confidence, non-repetitive DNA circles from 1150 out of 25 720 total MIC contigs in the current MIC assembly (1). While 604 of the 1150 MIC contigs have one high confidence circle annotation, three MIC contigs contain the highest density of eccDNAs detected, with 18 high confidence circle annotations per MIC contig (Supplementary Figure S3A). The stringent set of criteria used to call high confidence circles likely underestimates the actual number of eccDNAs present, suggesting that our method captured the most abundant eccDNAs present during rearrangement, rather than a comprehensive circle set.

**Figure 5.**
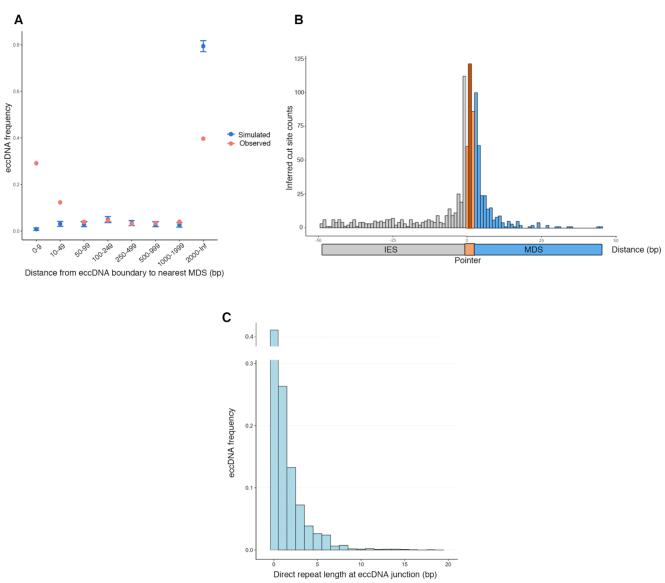
Characteristics of high confidence rearrangement-specific eccDNA. (**A**) For the 2432 high confidence eccDNA identified in mid-rearrangement, the distance to the nearest MDS boundary was measured and shown as red points. The distances are binned according to the values specified on the *x*-axis. The set of 2432 high confidence eccDNA were randomized 500 times across the MIC assembly and the blue points represent the mean frequency of each bin for the simulated data. The error bars on the simulated data represent three standard deviations. (**B**) The locations of 933 cut sites inferred from high confidence eccDNA that are within 50 bp of an MDS boundary that also have high confidence pointer annotations are shown. Each bar represents 1 bp, with the exception of the dark orange bar in the middle of the pointer, which represents all cut sites within variable length pointers. The distribution of cut sites inside IESs is significantly different from the distribution of cut sites inside MDSs (Kolmogorov–Smirnov test, *P*-value = 4.108 × 10^−15^). (**C**) The length of the direct repeats flanking circularly excised loci for the 2432 high confidence eccDNA are shown.

However, even this limited set of high confidence circles reveals intriguing patterns. We investigated the relationship between circles and MDSs, noting that circular DNA originates from both MDS rich and poor regions. While the median distance of eccDNAs from an MDS boundary is 255 bp, 41% of circles map within 49 bp of an MDS boundary, and 40% have an MDS boundary at a distance of >2000 bp or are found on MDS-lacking MIC contigs (Figure 5A). To compare to a simulated expected eccDNA distribution, we randomly shuffled the eccDNA annotations across the MIC assembly. In this randomized dataset we observed that eccDNA would rarely fall in close proximity to MDSs by chance, suggesting that the actual dataset is strongly enriched for eccDNA in MDS-rich regions (Figure 5A). On the other hand, eccDNAs that are at a distance of >2000 bp or derive from MDS-lacking MIC contigs are depleted in our dataset, compared to the random distribution (Figure 5A). We note that extensive MDS paralogy exists in the MIC, where an MDS is present in multiple copies throughout the MIC (50). Paralogous MDSs exhibit varying levels of similarity and may be omitted in the annotations. Therefore, the number of eccDNA that map far away from any MDS boundary may be an overestimate.

We also set out to classify the types of non-repetitive MIC-limited loci that give rise to the circles. For the circles that have both ends within 50 bp of an MDS boundary, we determined the type of eliminated sequence that would be removed via circularization. We found high confidence circles containing at least three different types of eliminated sequence: non-scrambled IESs that interrupt consecutive MDSs, which we expected to be excised as circles (3), but also scrambled IESs that map between non-consecutive MDSs and intergenic regions near chromosome breakage sites between MDSs for different MAC chromosomes. We do find that eccDNAs derive from non-scrambled IESs at much higher frequency than scrambled IESs (0.46 and 0.04%, respectively, *P*-values < 2 × 10^−16^, chi-squared test) (Table 2). Additionally, 86 eccDNAs may carry MDSs, as indicated by the chimeric junction read mappings that flank complete MDSs. Six of these eccDNA bear MDSs that map within an IES of another MAC locus.

**Table 2.** Categories of non-repetitive MIC-limited loci giving rise to eccDNA

	No. of MAC contigs	No. of eliminated sites in the MIC	No. of eliminated sites containing eccDNA	Percentage of eliminated sites containing eccDNA
Non-scrambled	15 680	119 613	555	0.46
Scrambled	2818	10 151	4	0.04
Intergenic		16 846	17	0.10

The smallest high confidence circle we detected is 78 bp long (Supplementary Figure S3B), which is just above the minimum length required for circularization of double stranded DNA (51,52). The median length of high confidence eccDNA is 616 bp, with a peak ∼400 bp (Supplementary Figure S3B), slightly longer than what was reported in mammalian tissues and cell lines (35). However, we note that the peak eccDNA size detected here is similar to the insert size of the Illumina libraries that were sequenced, suggesting that the circle size distribution may be heavily biased by the size selection step of the library preparation. The smallest IES that is circularly excised is 57 bp, excluding pointers, and gives rise to an 80 bp eccDNA containing the IES together with some flanking MDS sequence.

### The eccDNA junctions are imprecise and not at pointers or extended cryptic repeats

The junction sequences identified in high abundance eccDNA (with multiple junction reads) suggest heterogeneous eccDNA formation and inferred cut sites clustering near pointers. Inferred cut sites are based on the coordinates of chimeric junction read alignments, as shown in Figure 2C. Analysis of 933 inferred cut sites for high confidence eccDNAs whose circular junctions map within 50 bp of an MDS boundary revealed the presence of inferred cut sites within pointers (29%), as well as within IES (39%) or MDS sequence (32%) (Figure 5B and Supplementary S3C). The cut sites inside of IESs and MDSs have significantly different distributions, with cut sites within IESs often further from pointers than those within MDSs. Within the MDSs, the mean and median distances from inferred cut site to MDS boundary is 4.75 and 2 bp, respectively, whereas within IESs the mean and median distance for cut site is 13.8 and 6 bp, respectively (*P*-value = 4 × 10^−15^ by two-sample Kolmogorov–Smirnov test) (Figure 5B). These results are surprising, given that most MDS–MDS junctions map within coding regions and that mature molecules at the end of rearrangement appear to contain precise junctions.

In order to gain insight into the repair process involved, we also investigated how often direct repeats that *differ* from the actual pointers (i.e. *cryptic pointers*) flank the circularization site. We find that 41.9% of high confidence circles do not have cryptic pointers immediately flanking the site of circularization, suggesting that the circularization of these loci is not homology-dependent at the junction (Figure 5C). Only a small subset of eccDNA (1.2%) utilize *bona fide* pointers for recombination. For the remaining cases that demonstrate recombination at cryptic pointers (56.9%), the majority contain AT-rich direct repeats <3 bp, which is not long enough to suggest homology-dependence (Supplementary Figure S3D). We also recovered some cases of eccDNA (17.3%) that suggest recombination at longer (3–18 bp) cryptic pointers (Supplementary Figure S3D).

### Validation of rearrangement-specific eccDNA

**Figure 6.**
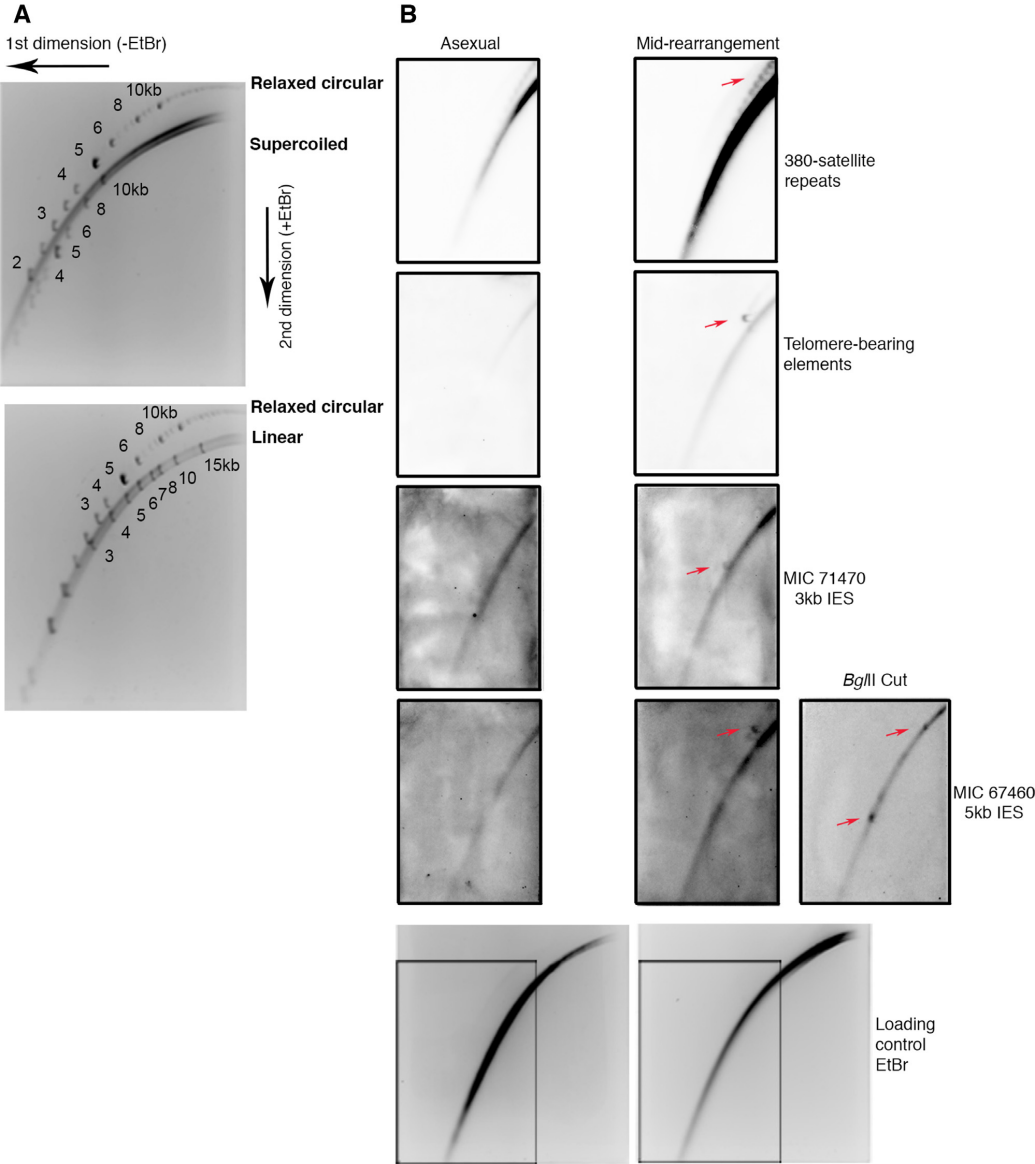
Validating and further investigating the circular conformation and topology of *bona fide* eccDNA using 2D agarose gel electrophoresis. (**A**) Whole-cell DNA from asexually growing cells was separated on a 2D agarose gel with spiked-in relaxed circular, supercoiled circular and linear DNA standards showing separation of topologically different DNA molecules. Numbers represent size of the spike-in standards in kb. (**B**) Whole-cell DNA during asexual growth and rearrangement was separated on a 2D agarose gel and coupled to Southern blotting with probes specific for 380 nt satellite repeats, TBEs, a 5 kb and 3 kb IES that were annotated to contain high confidence circles. The same gel was stained with ethidium bromide to show equal loading of DNA. Membranes were stripped and reprobed sequentially for the four loci. Whole-cell DNA during mid-rearrangement was cut with BglII, which has a single target restriction site in the 5 kb IES circle in parental strain JRB310, leading to a 5 kb linear fragment. The other parental strain, JRB510, has two BglII restriction sites producing a 1.8 kb linear fragment that contains the probe hybridization target site. The cut DNA was separated on a 2D gel similar to the previous uncut samples and probed for the 5 kb IES circle. Red arrows mark the spots at the expected sizes for the two parental strains or overlapping the arc of linear DNA.

To validate and further investigate the circular conformation and topology of excised TBEs during rearrangement, we used Southern hybridization with 2D agarose gel electrophoresis, to separate DNA by size and structure, allowing linear, relaxed circular and supercoiled circular DNA to be visualized as separate arcs (Figure 6A). In order to validate the separation of circular DNA from *Oxytricha*’s linear genomic chromosomes and from long, rearranging MIC precursor fragments, we first probed for an abundant class of MIC-specific 380 bp satellite repeats (1,53). Satellite repeats are prone to circularization in a wide range of model organisms such as *Xenopus*, mouse, plants and humans (27,54–56). Southern hybridization with a probe specific to the 380 bp satellite repeat in *Oxytricha* identified two arcs on a 2D gel, with the bottom continuous arc representing linear genomic chromosomes and the top arc representing circular multimeric repeats of various lengths, present during both asexual growth and rearrangement (Figure 6B). Similar to 380 bp satellite repeats, Southern hybridization with a probe specific for TBEs identified a spot off the arc of linear genomic chromosomes representing circular TBEs (Figure 6B). The absence of a strong signal on the arc of linear molecules corresponding precisely to 4 kb suggests that the majority of excised TBEs are in circular form, while the continuum of linear molecules may represent variable length rearrangement intermediates that contain unexcised TBEs or high molecular weight MIC DNA that was sheared during pipetting. Unlike 380 bp satellite repeats, we could detect the presence of circularized TBEs only during rearrangement, which is accompanied by an increase in MIC DNA copy number. The migration of both 380 bp satellite repeats and TBEs above the arc of linear genomic chromosomes suggests that these are nicked, open circles. Thus, we conclude that repetitive MIC-limited regions give rise to *bona fide* eccDNA in *Oxytricha*.

Having validated the circular excision of TBEs using Southern hybridization with 2D gels, we stripped and probed the membranes with two IES sequences containing high confidence circle annotations. We identified a spot off the arc of linear chromosomes that may contain a continuum of variable length rearrangement intermediates bearing the particular IES, for both of the probed circles, exclusively in mid-rearrangement, indicating that these 3 and 5 kb IESs are also excised as *bona fide* open circles similar to TBEs, but at lower abundance, as expected (Figure 6B). Cleavage of DNA with BglII, which has restriction sites within the high confidence 5 kb IES circle, abolishes the hybridization signal that was above the arc of linear chromosomes (Figure 6B), further validating this eccDNA. One of the parental strains has a single BglII restriction site inside this IES, leading to a 5 kb linear fragment, whereas the other parental strain has two BglII restriction sites producing a 1.8 kb linear fragment that contains the probe hybridization target site.

**Figure 7.**
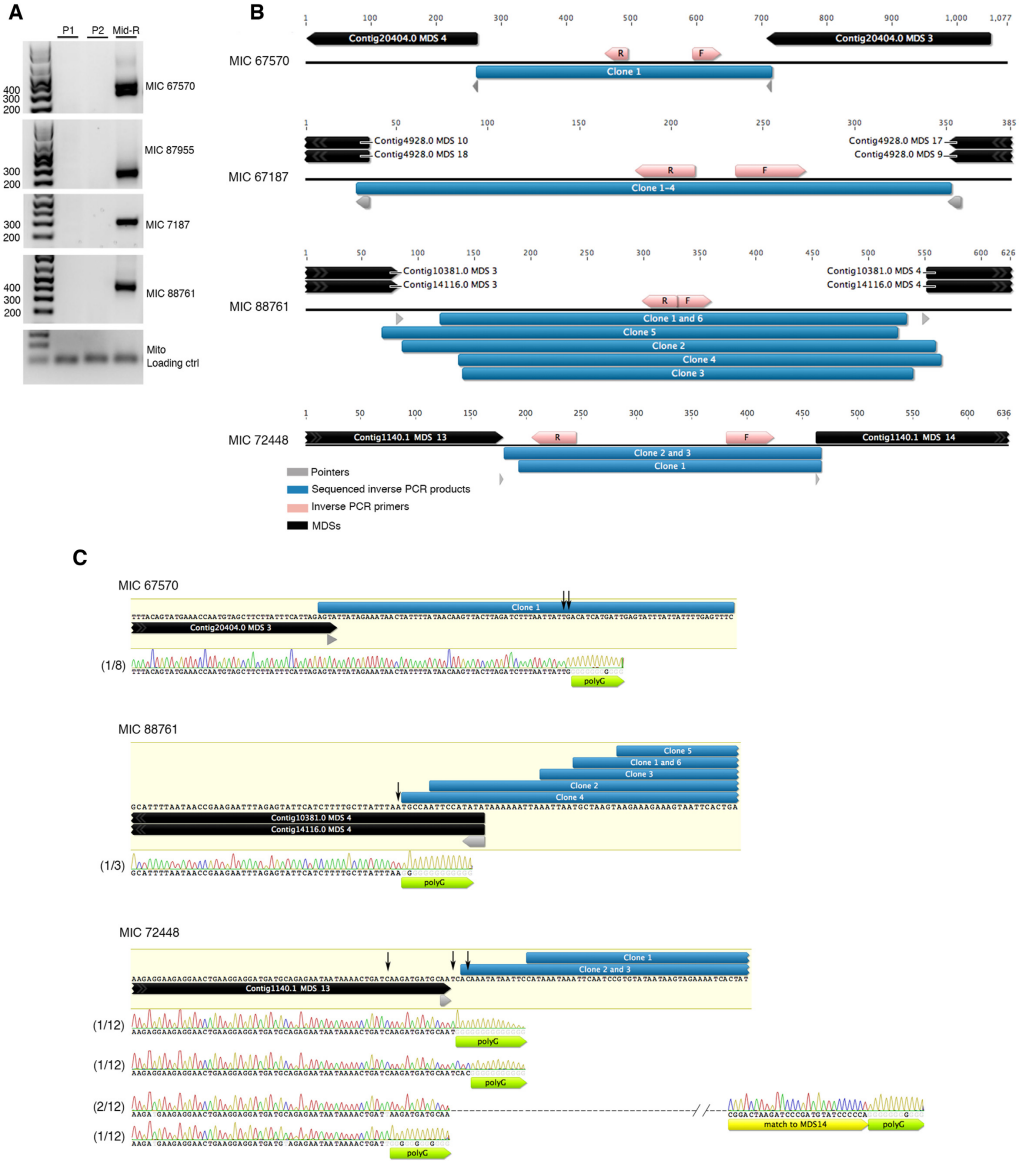
Inverse PCR validation of rearrangement-specific eccDNA. (**A**) Inverse PCR using primer pairs that point away from each other were used to validate circularly excised loci identified by circular junction reads (MIC67570 and MIC87955) and 9 bp duplications (MIC 67187 and MIC 88761). P1 and P2 represent whole-cell DNA from asexually growing parental strains. Mid-R represents whole-cell DNA from cells during mid-rearrangement. Mitochondrial primers (Mito) were used to show equal loading of whole-cell DNA. (**B**) Clones obtained from inverse PCR products amplified using primer pairs that point away from each other (shown as pink arrows) were sequenced and are shown as blue bars. MDS annotations and pointers flanking IESs are shown as black and gray arrows respectively. (**C**) Terminal transferase treatment of genomic DNA during mid-rearrangement suggests variable 3′ DNA breaks at MDS boundaries for three loci. Green arrows indicate where the dGTP tracts are added by the terminal transferase. The black vertical arrows indicate where the 3′ breaks map, as determined by the dGTP tracts. MIC 67570 contains an encoded G at the site of G-tailing, which leads to 1 nt ambiguity in mapping the precise 3′ breakpoint, denoted by two black arrows shown side by side. One clone for MIC72448 indicates polyG addition downstream of the MDS 13–14 junction (yellow arrow indicates partial match to MDS14), suggesting that this clone likely originates from either the old degrading MAC or a partially rearranged molecule. The total number of clones sequenced and the number of clones that correspond to the mapped ends are noted before each electropherogram.

Inverse PCR provided another method to validate eccDNAs in non-repetitive MIC-limited loci. Inverse PCR using outward-pointing primers for four candidate eccDNAs amplified a product exclusively during rearrangement, and not in the parental cells (Figure 7A); two cases are high confidence annotations based on junction reads and two are based on the signature 9 bp duplication in read pairs (Figure 4C). The sequenced PCR amplicons for these loci, together with another IES with high Circulome-seq read coverage, validate the presence of eccDNA and demonstrate the imprecise and heterogeneous circular elimination of IESs with inferred cut sites in the vicinity of pointers (Figure 7B). Thus, in addition to having captured eccDNA genome-wide, we also confirmed the presence of eccDNA by 2D agarose gel electrophoresis and inverse PCR.

To map the cut sites on one strand that give rise to eccDNAs, we used terminal transferase to add dGTP tracts to the 3′ DNA breaks at MDS boundaries (Supplementary Figure S4). As expected, we find evidence supporting the presence of variable cut sites at MDS boundaries for the three loci that we tested (Figure 7C). In addition to mapping 3′ DNA breaks inside IESs, as in MIC 67570 (1 out of 8 clones) and MIC 72448 (2 out of 12 clones), we also find evidence supporting 3′ DNA breaks inside MDSs, as in MIC 88761 (1 out of 3 clones) and MIC 72448 (1 out of 12 clones). Furthermore, the sequenced 3′ DNA break in MIC 88761 precisely matches the sequenced eccDNA junction clone (Figure 7C). We also recovered clones that contain mature MDS-MDS junctions in MIC 72448 (2 out of 12 clones) which suggests that this approach may also capture DNA breaks in the degrading, parental MAC, or in partially processed molecules during rearrangement (Figure 7C). The remaining clones were the result of misannealed locus-specific primers and map to other MDSs. The fast kinetics of MDS-MDS ligation may prevent the capture of abundant breaks at MDS boundaries. Even though the cut sites inside MDSs may also originate from the degrading MAC, the cut sites inside IESs most likely derive from the rearranging MAC, providing evidence that corroborates with our observations of heterogeneous and imprecise eccDNA boundaries in the Circulome-seq dataset.

### Non-coding RNA transcripts from eccDNA

**Figure 8.**
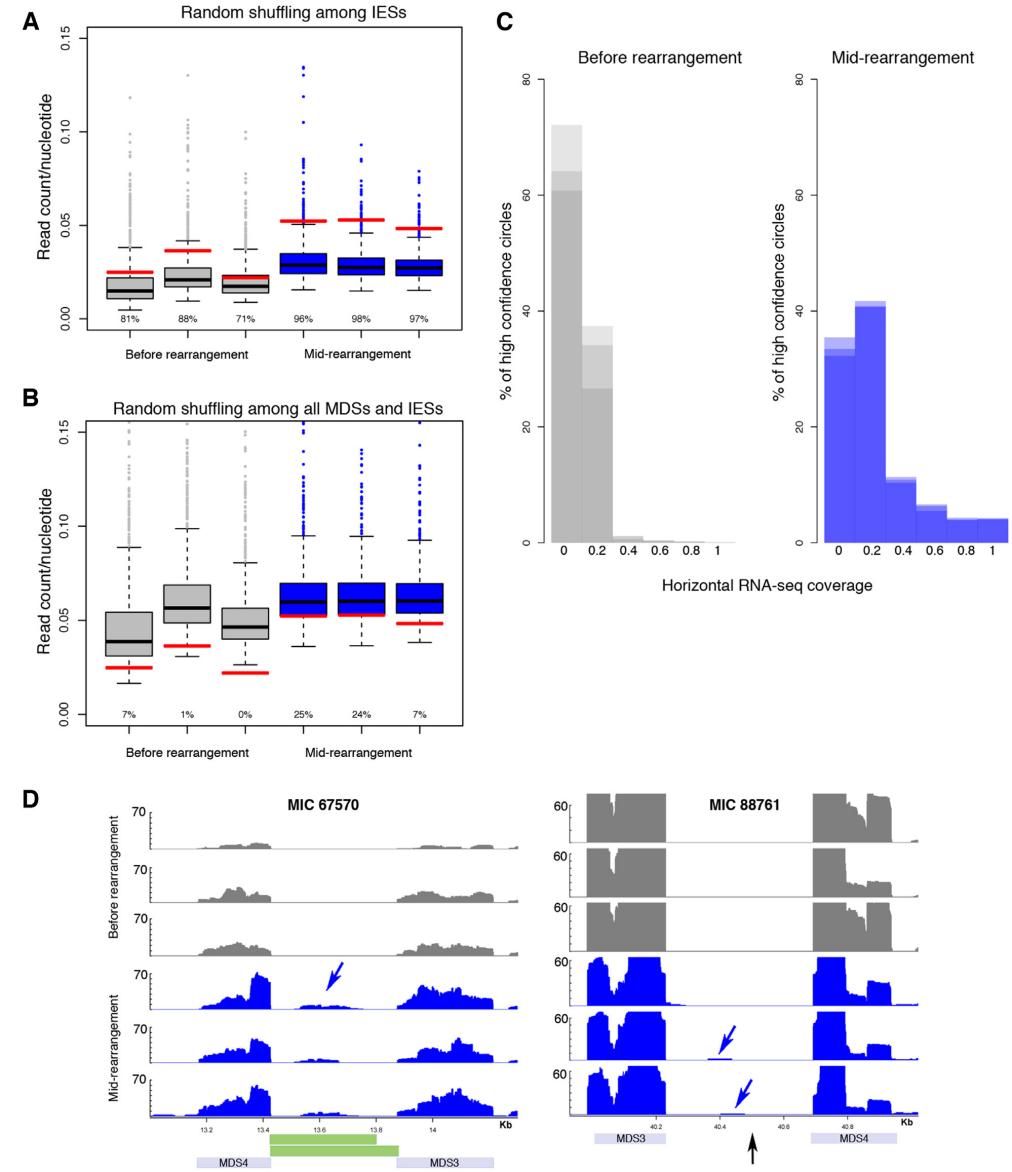
Rearrangement-specific transcription from high confidence eccDNA. (**A**) Red lines show mean RNA-seq read counts normalized by circle length for 2432 high confidence eccDNA. The 2432 intervals were randomly shuffled 1000 times among all IESs, genome-wide. The boxplots indicate the simulated distribution for the 1000 randomizations. Gray boxplots represent RNA-seq data collected in triplicate before early rearrangement, 12 h post-mixing. Blue boxplots represent RNA-seq data collected in triplicate in mid-rearrangement. Values under the boxplots represent the percentages of dots in the randomized set that fall below the observed mean RNA-seq read counts for the annotated eccDNA. (**B**) The 2432 eccDNA intervals were randomly shuffled throughout the whole MIC assembly, including MDSs that contain gene sequences. The boxplots indicate the simulated distribution for the 1000 randomizations throughout the whole MIC assembly. (**C**) Horizontal RNA-seq read coverage was calculated as the fraction of each eccDNA across its length with at least one read mapped. Gray histogram represents three RNA-seq replicates collected prior to rearrangement. Purple histogram represents three RNA-seq replicates collected at mid-rearrangement. (**D**) Genome tracks for two validated eccDNA showing RNA-seq read coverage in the region that circularizes. For MIC 67570, eccDNA annotations inferred from junction reads are indicated by green rectangles. For MIC 88761 the IES that has a read pair with the signature 9bp duplication is marked with a black arrow. Blue arrows point to RNA-seq read coverage in IESs that give rise to validated eccDNA.

To query if the circularly excised MIC-limited sequences might be more than elimination byproducts and to test the hypothesis that they may provide templates for a rearrangement-specific non-coding RNA pathway, similar to iesRNAs in *Paramecium* (31), we asked whether the high confidence eccDNAs are transcribed. RNA-seq data collected at mid-rearrangement show that IESs that give rise to eccDNA have significantly more RNA-seq read counts compared to a random distribution among all IESs (97% of the randomized interval sets contain fewer reads than eccDNAs) (Figure 8A). In samples collected at the 12 h time point before rearrangement, there is no significant difference between RNA-seq counts within circles versus random intervals (Figure 8A). This suggests that there is rearrangement-specific production of ncRNAs from IESs that give rise to high confidence eccDNA. When the high confidence circle intervals are shuffled across the whole MIC genome, this effect is no longer observed, suggesting that eccDNA-specific transcription levels are lower than the levels of genic transcription (Figure 8B). We next looked at horizontal RNA-seq coverage, the fraction of each circle along the length that is covered with at least one read. While 65.7% of high confidence eccDNA have no RNA-seq coverage prior to early rearrangement, in mid-rearrangement only 33.7% have no coverage. Moreover, in mid-rearrangement 25.2% of eccDNA have >20% coverage and 4.1% have >80% coverage (Figure 7C). We conclude that there is rearrangement-specific transcription of a subset of high confidence eccDNA molecules, although we cannot exclude the possibility that all eccDNA are transcribed to produce ncRNAs. In Figure 8D we show examples of RNA-seq reads derived from high confidence eccDNA loci, demonstrating high and low levels of eccDNA transcription, specifically in mid-rearrangement (Figure 8D).

**Figure 9.**
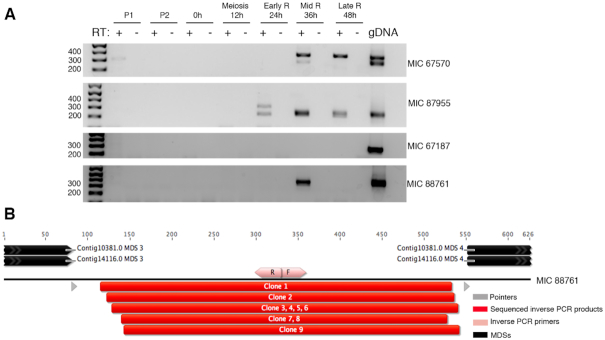
Inverse RT-PCR suggests transcription across eccDNA junctions. (**A**) Inverse RT-PCR using primer pairs that point away from each other were used to show transcription from eccDNA in asexual cells and throughout rearrangement. P1 and P2 represent asexually growing parental strains and ‘gDNA’ represents conventional inverse PCR using mid-rearrangement whole-cell DNA as template. Control reverse transcription reactions lacking reverse transcriptase are represented by RT-. (**B**) Clones obtained from inverse RT-PCR using primer pairs that point away from each other (shown as pink arrows) were sequenced for MIC 88761 and are shown as red bars. MDS annotations and pointers flanking IESs are shown as black and gray arrows respectively.

To specifically query transcription across circle junctions, we used inverse PCR on cDNA templates generated using random hexamers from total RNA. This method recovered circular junctions exclusively during rearrangement for three out of four high confidence eccDNA, consistent with transcription across the circle junction from at least a subset of circularly excised loci (Figure 9A). Control experiments without reverse transcriptase did not recover any products containing circular junctions. Peak transcription appears to occur mid-rearrangement, with lower levels detected in early- and late-rearrangement, recapitulating the temporal pattern for eccDNA production that we observed in the Circulome-seq data. Furthermore, the sequenced amplicons display a similar pattern of heterogeneity and imprecision at the junctions to those observed in inverse PCR using DNA as template (Figures 7B and 9B), consistent with the hypothesis that the circularly eliminated eccDNA are transcribed. Most sequenced eccDNA and RT-PCR clones do not perfectly align, with one exception: an inferred cut site in eccDNA clone 3 (Figure 7B) that precisely matches RT-PCR clone 7 and 8 (Figure 9B).

**Figure 10.**
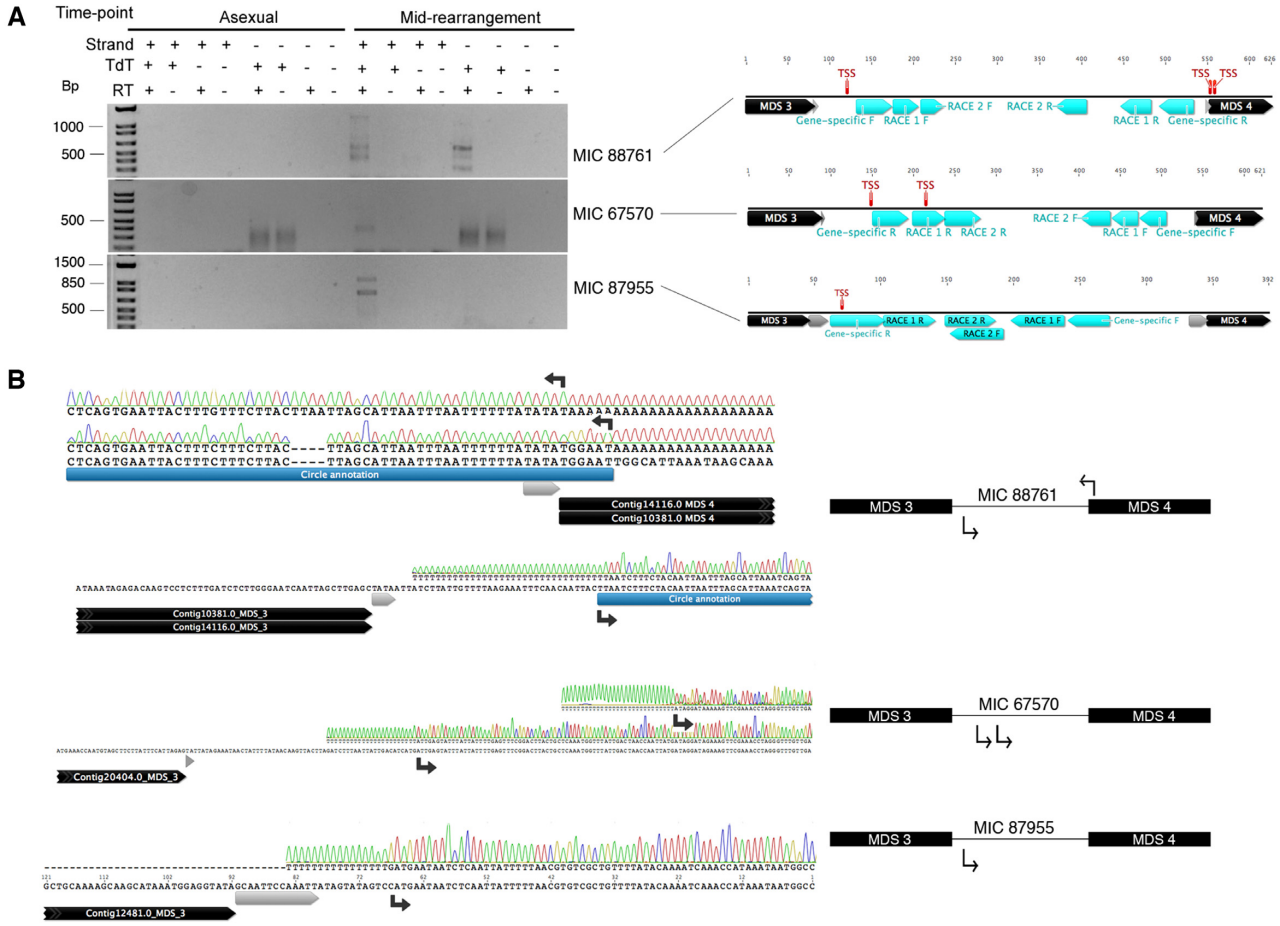
5′- RACE reveals TSS sites within eccDNA. (**A**) 5′-RACE products for three validated eccDNAs are resolved using agarose gel electrophoresis. Control A-tailing reactions lacking terminal transferase are represented by TdT-. Control reverse transcription reactions lacking reverse transcriptase are represented by RT-. The directionality of transcription was determined using two different sets of primers for each candidate and is represented by + or – strand. The locations of the three locus-specific primers used for each direction is represented by cyan arrows. The sequenced 5′-RACE products that revealed a TSS in the vicinity of the pointers are shown as red lines. Pointers are represented by gray arrows. (**B**) The electropherogram for polyA-containing sites are shown. Black arrows show the location of the TSSs and their direction as suggested by the sequence of the 5′-RACE product.

To further exclude the possibility that the transcripts we detected are due to read-through transcription of long, MDS-containing DNA molecules undergoing rearrangement and to demonstrate that the transcripts are eccDNA-specific, we used 5′-RACE to characterize TSSs within eccDNA at nucleotide resolution. We targeted the three eccDNA molecules for which we detected transcripts containing circular junctions via inverse RT-PCR (Figure 9B). While we detect TSSs in both directions for eccDNA in MIC 88761, we detected TSSs in only one direction for eccDNAs from MIC 67570 and MIC 87955 (Figure 10A). Furthermore, most TSSs that we detected cluster near the pointers, where eccDNA boundaries also reside. Two of the TSSs for MIC 88761 precisely overlap with eccDNA boundaries inferred via inverse PCR from whole-cell DNA (Figure 7B and 10B). Therefore, we conclude that eccDNA-specific TSSs appear to cluster near the circle junctions, and this would enable bidirectional eccDNA transcription.

## DISCUSSION

Genome-wide capture of circular DNA allowed us to recover and sequence a wave of hundreds of circularly eliminated TBE transposons, together with thousands of distinct, non-repetitive germline-limited loci, during programmed genome reduction as part of *Oxytricha* nuclear development. The circularly eliminated non-repetitive sequences we recovered derive from mostly non-scrambled IESs, and rarely from scrambled IESs and intergenic regions between MDSs that map to different MAC chromosomes. We present three lines of evidence to support the circular excision of these eliminated sequences: (i) Circular DNA enrichment coupled to deep-sequencing (ii) Southern hybridization of 2D agarose gel electrophoresis and (iii) inverse PCR analysis.

There is great diversity in the structural features and removal of IESs in other ciliate model systems. *Paramecium* IESs are usually found in coding regions and flanked by 2 bp TA pointers that precisely join the adjacent MDSs (57). A 4 bp staggered cut centered on the TA direct repeat is followed by resection of the 5′ nucleotide and filling in on both sides of the palindromic TA to form the eccDNA junction (58). *Euplotes* IESs are also flanked by TA direct repeats, but the model for IES elimination in *Euplotes* suggests that the circularized IES contains both copies of the TA direct repeat, separated by a variable heteroduplex region derived from the sequences flanking both sides of the IES, based on strand-specific PCR and sensitivity to S1 and Bal-31 nucleases (15,16). In contrast, in *Tetrahymena*, most IESs are flanked by 1–8 bp variable direct repeats that recombine imprecisely and consequently, most IESs map to non-coding regions (59) where imprecise excision can be tolerated. *Tetrahymena* IES excision appears to produce both linear and circular molecules (60–62). *Oxytricha* therefore shares the feature with *Tetrahymena* of harboring direct repeats flanking non-scrambled IESs that vary in sequence and length (average length for non-scrambled and scrambled pointers, 5 and 11 bp, respectively (1)). However, like *Paramecium*, they generally interrupt ORFs and must be removed precisely to form functional genes in the MAC (2).

In support of the circular elimination of TBEs during genome rearrangement (first case described in Williams *et al.*(17)) (Figure 1B) we found hundreds of chimeric reads spanning circular TBE junctions in mid-rearrangement (Figure 3A and Table 1), all suggesting the precise excision of TBEs with one copy of the TSD on the removed circle, while the other copy is left behind at the site of excision from the genome. At our higher resolution, we found two other junction motifs, GANTA and GANTG, in addition to the previously identified GANTC motif (17) for TBE excision, where ANT is the TSD (Figure 3B). Given that the TIR sequence internal to the TSD is highly conserved and the presence of a C, T, G bias at the nucleotide immediately flanking the TSD (Figure 3C), similar to the bias at the circular junction motif, we speculate that TBEs are excised via longer overhangs than the previously proposed 3 bp staggered cuts. Excision via a 5 bp staggered cut centered on ANT would account for the variable circle junction motif and lead to the formation of a heteroduplex junction, similar to Tec element and IES removal in *Euplotes* (15,16). However, we cannot exclude other post-excision processes, such as resection and base addition at the cut site prior to circularization, since these could also account for the observed variability.

For circles in non-repetitive MIC-limited loci, while 40% of circles map far away from MDS boundaries (Figure 5A), among circles that map close to MDSs we generally found evidence for the circularization of non-scrambled IESs, independent of homologous recombination between the pointers flanking non-scrambled IESs. This differs from the recombination between adjacent MDSs (63–65). To our surprise, we also found evidence for low levels of circularization of scrambled IESs, even though an intervening IES between scrambled MDSs is not flanked by matching pointers (Table 2). However, the set of high confidence eccDNA is significantly enriched in non-scrambled IESs, supporting their primary elimination by this pathway. We also find 17 cases of circularization of intergenic loci between MDSs that map to different MAC chromosomes. These cases all lie on or near chromosome breakage sites, as determined by neighboring terminal MDSs. Maternal RNA templates may facilitate looping in regions to be eliminated, which would lead to the preferential circularization of non-scrambled IESs.

In contrast to the uniform junction reads that suggest cut sites at a precise location for elimination of TBE transposons, both our genomic survey and inverse PCR suggest that circularization of non-repetitive MIC-limited loci occurs at variable and imprecise junctions (Figures 4C, 5B and 7B), even though these junctions frequently lie within ORFs. Inferred cut sites nevertheless cluster near the pointers at MDS-IES boundaries and more often occur within a pointer, but not precisely at its boundaries. Furthermore, there is significantly less constraint for how far the cut site might extend within an IES compared to an MDS (Figure 5B) suggesting that it may be easier to remove residual IES sequence at MDS-MDS junctions, a consequence of cut sites within IESs, than it would be to fill-in lost MDS sequence in coding regions, if cut sites occur within MDSs; however we do not have information on the opposite strand or the presence of an overhang. Indeed, during rearrangement we capture broken 3′ DNA ends near three MDS boundaries neighboring IESs that give rise to eccDNA. Consistent with our Circulome-seq study, the breaks that cluster around pointers map within IESs as well as MDSs (Figure 7C).

The above observations related to MIC-limited non-repetitive eccDNA are consistent with a general model in which widespread DNA cleavage occurs preferentially outside of regions protected by *Oxytricha* piRNAs that mostly mark MDSs, with sparser mapping to non-coding subtelomeric regions and almost no mapping to IESs (13,66). Such a coarse method of eliminating DNA may be helpful in reducing the sequence space that needs to be searched before more complex rearrangements occur, but it also implicates a need for maternal RNA template-guided error correction and repair (11,12,67).

The combination of aberrant circularization of non-repetitive MIC-limited loci at variable and imprecise junctions, together with occasional circular elimination of scrambled IESs, could lead to errors in rearrangement. For example, the incorrect removal of sequence between cryptic pointers was observed both in Mollenbeck *et al.* (67), which captured transient errors during rearrangement in WT cells, and in Nowacki *et al.* (11), which used RNAi to deplete specific template RNAs that guide rearrangement. RNAi against two genes, led to several cases of aberrant MDS-MDS junctions between non-scrambled MDSs as well as scrambled MDSs that are joined by cryptic pointers (11). Notably, these maternal RNA templates are also capable of guiding DNA base substitutions in the vicinity of pointers. An alternative model that accounts for the imprecision observed at circle junctions may involve the linear excision of IESs, followed by end resection and circularization, resulting in heterogeneous circular isoforms. Any model in which cut sites occur strictly within IESs would also require resection into the MDS on either strand to produce either the observed circular IESs containing partial MDS sequence or inferred cut sites in MDSs.

The machinery responsible for the excision and circularization of eccDNA during rearrangement in *Oxytricha* is unknown. The majority of loci giving rise to high confidence eccDNA are not flanked by substantial microhomology (Figure 5C), hence the removal of such MIC-limited loci must be independent of homologous recombination at adjacent repeats. A pathway similar to canonical non-homologous end joining (NHEJ) might be responsible for the repair of the circle junctions. We find that the *Oxytricha* genome (2) harbors a complete set of core NHEJ machinery, including Ku70, Ku80, Xrcc4, Lig4 and DNAPKcs, but it is unknown whether this machinery is essential for circular excision in *Oxytricha* as it is in *Paramecium*, whose circular IES excision requires Lig4 and Ku70/80 (68,69). However, a subset (18.6%) of high confidence eccDNAs in this study, recovered by chimeric junction reads, are flanked by 3–18 bp of microhomology (including those eccDNAs that recombined at *bona fide* pointers) (Figure 5C). Future studies may investigate whether there are different classes of circularly removed sequences, with different characteristics and machinery responsible for their elimination. Previous work showed that the TBE-encoded transposase is essential for the processing of high molecular weight DNA to MAC DNA during genome rearrangement (70), suggesting the possibility of transposase-mediated cleavage of non-repetitive MIC-limited DNA, although our study suggests that such cleavage is less precise than cleavage of transposons, themselves. Hence, there may be a suite of different nucleases operating on MIC-limited DNA, with differing substrate requirements.

The fact that this study captured only a minority of non-repetitive MIC-limited regions as eccDNA may be a technical limitation of our sequencing approach, although we cannot exclude the possibility that only a subset of non-repetitive MIC-limited loci form circles. Circularization of eliminated sequences might prevent excised sequences from reintegrating elsewhere, which would compromise genome integrity, as suggested by Kapusta *et al.* (68) or it may permit robust transcription of IESs, possibly supporting the production of other classes of non-coding RNAs similar to the multiple classes of small RNAs (iesRNAs or scnRNAs) that mark deleted DNA in *Paramecium* and *Tetrahymena* (31,71).

Indeed, the appearance of a specific peak of eccDNA transcription at mid-rearrangement, among hundreds of high confidence cases (Figures 8 and 9), suggests that production of circularly excised IESs may be more than rearrangement byproducts destined for degradation. The observation that eccDNA-specific TSSs cluster around circle boundaries (Figure 10) raises the possibility that transcription is primed at nicked eccDNA junctions. Transcription initiation at nicked circular double-stranded DNA has previously been observed in the absence of canonical promoters (72). This is consistent with the 2D-agarose gel electrophoresis that suggests the presence of mostly relaxed eccDNA during rearrangement. Therefore, it is possible that ncRNA synthesis may be initiated at nicked eccDNA junctions or junctions that were not fully ligated.

Recently, transcription from eccDNA has been demonstrated in mammalian cells. These transcripts are processed into small regulatory RNAs that can modulate endogenous gene expression (30). *Oxytricha* possesses many Piwi paralogs (13). Hence it is possible that the IES eccDNA transcripts are precursors to a novel Piwi-dependent small RNA pathway in *Oxytricha* that mark sequences for deletion, in contrast to Otiwi1-dependent piRNAs (13) that mark MDSs for retention. However, widespread transcription during rearrangement could also account for these observations. Future work should investigate the possible roles of eccDNA transcripts in programmed genome rearrangement.

Unlike previous predictions suggesting precise circular excision of non-scrambled IESs via homologous recombination at direct repeats, here we show evidence for circular excision of non-repetitive MIC-limited loci via non-specific cleavage in the vicinity of MDS-IES boundaries within both non-scrambled and scrambled IESs, as well as intergenic loci that contain chromosome breakage sites. In contrast, TBE transposable elements are precisely removed as circular molecules. Our model suggests non-specific and widespread cleavage by one or more nucleases within non-repetitive MIC-limited loci, leading to widespread circular excision of germline-limited DNA, as part of a complex cascade of events leading to the restoration of genome integrity in *Oxytricha*’s production of a new macronucleus.

## DATA AVAILABILITY

Circulome-seq reads are available through NCBI Short Read Archive (SRA) with the following accession number: PRJNA526276. RNA-seq reads are available through European Nucleotide Archive (ENA) with the following accession number: PRJEB32087. All custom scripts used in the analysis of Circulome-seq will be made available upon request.

## Supplementary Material

gkz725_Supplemental_File

## Notes

Present address: Rafik Neme, Department of Chemistry and Biology, Universidad del Norte, Barranquilla, Atlántico, Colombia.
