# Supplementary material for: Programmed genome rearrangements in Oxytricha produce transcriptionally active extrachromosomal circular DNA
Source: Nucleic Acids Res. 2019 Aug 28;47(18):9741–60. doi: 10.1093/nar/gkz725 (PMC6765146; doi:10.1093/nar/gkz725)

Supplemental Table 1: PCR primer sequences

**Two-dimensional Southern blotting:**	
TBE	5'-GTAACCTGGAGTACCAAGAACC-3'<br>5'-TGCCCTTTAGAAGTAGTCGTAATC-3'
380bp (76)	5'-TGAGATGACCTGGAATTAAGCA-3'<br>5'-TATATCTGGTGTTCCGGTGTCA-3'
MIC_67460	5'-GTCAAAAAGTAGAACAGCTAATCATCTTTC-3'<br>5'-AAAGTAGGTCTAGTCCAGTCAG-3'
MIC_71470	5'-CAGCATTACCAGTTGACTAAATATTC-3'<br>5'-GTCATGAGGAGTAGGCAGAC -3'
**Inverse PCR validation of eccDNA:**	
MIC87955	5'-CCATTATTTATGGTTTGATTTTGTATAAAACAGCGACACG-3'<br>5'-CCATTTCTCAACTATATAAGCCAAGATTTTAATCACAGAG-3'
MIC67570	5'-TTAGTCAATAAACCATTTGAGCAGTAAGTCCGAAACTC-3'<br>5'-AGCACGGGATACATGTCTGAAGCTGTTATG-3'
MIC88761	5'-AGACATTAGAGTGATTAGGTACTTACTTC-3'<br>5'-TAGATAACCACTGACTGAAGATTCTTATC-3'
MIC67187	5'-ACAATATTCATTTCCATTAATAGATTAATTTTATGTG-3'<br>5'-TCTATTCGGTAAAATCATTTAAGAAATAGGTC-3
MIC72448	5'- TGATTTTCCTGAGAATAGTCTTTTATTAAGTGATATAGTTTC-3'<br>5'- ACTCTACATAGTGATTTTCTACTTATTATACACGGATTG-3'
**Mapping 3' DNA breaks:**	
Qt (50)	5'CCAGTGAGCAGAGTGACGAGGACTCGAGCTCAAGCGGGGGGGGGGGGGGGGG-3'
Qo (50)	5'-CCAGTGAGCAGAGTGACG-3'
Qi (50)	5'-GAGGACTCGAGCTCAAGC-3'
A(C) (62)	5'GATCTCATGCTGGAGTTCTTCGCCAAGTCCCCCCCCCCCCCCCC-3'
A (62)	5'-GATCTCATGCTGGAGTTCTTCGCC-3'
MIC72448 first	5'-ACGAGTACGATCATCTCTACGATTGGGTGATG-3'
MIC72448 nested	5'-ACAACCAGCACCTTGAAAACATATTCACAGTGGAG-3'
MIC67570 M3 boundary first	5'-AATGGATACGCTGCTTCAATAATTGCTCTCTTATTTAGC-3'
MIC67570 M3 boundary nested	5'-AGCTTTATAGTATATATGCTATTTTCCATACTTGCTTCTG-3'
MIC67570 M4 boundary first	5'-TCCTCCTCTTCATCTTTTAATTCCTAATAGTTAGAG-3'
MIC67570 M4 boundary nested	5'-ACTATGAGCTATTTATTTTGAAATTGTGTTGTTTCTGTACTC-3'
MIC88761 M4 boundary first	5'-ACTAACGAAGTCAATGTATAATTAGCTTGTGATTAAATTAGC-3'
MIC88761 M4 boundary nested	5'-ATCAGGCTCATCTTCTTCTCCAGGTACTGGTC-3'

**qPCR primers:**	
pUC19	5'-CTACATACCTCGCTCTGCTAATC-3'<br>5'-GCGCCTTATCCGGTAACTATC-3'
Mito	5'-CTCGATGTCGGCTCATCATATC-3'<br>5'-ACGTTCTAAACCCAGCTCAC-3'
**5' RACE:**	
67570_GSP_F	5'-GGAAATTAATGTTAAGGGGAGGAAGCTGAC-3'
67570_RACE_1_F	5'-CCCGACTTCTAAACCAGATGTTACAAGTG-3'
67570_RACE_2_F	5'-CCTCGCCAAACAATTCTATCATCTAAACAAGCG-3'
67570_GSP_R	5'-GAGTATTTATTATTTTGAGTTTCGGACTTACTGCTCAAATG-3'
67570_RACE_1_R	5'-GACTAACCAATTATGATAGGATAGAAAGTTCGAAACC-3'
67570_RACE_2_R	5'-GGGTTTGTTGATATTTTCTCTTGTAAAAGAAAGAATATTCC-3'
87955_GSP_F	5'-CGATCTATGCGAGCTATGATTGCATATCCC-3'
87955_RACE_1_F	5'-CTGTGATTAAAAATCTTGGCTTATATAGTTGAGAAATGG -3'
87955_RACE_2_F	5'-CTAAATCTCTATGCCTACATTATTGATACTTGTACTATC-3'
87955_GSP_R	5'-GTATAGTCCATGAATAATCTCAATTATTTTTAACGTGTCG-3'
87955_RACE_1_R	5'-CTGTTTTATACAAAATCAAACCATAAATAATGGCCGTG -3'
87955_RACE_2_R	5'-CAAAGATAGTACAAGTATCAATAATGTAGGCATAGAG-3'
88761_GSP_F	5'-CAATTAATTTAGCATTAAATCAGTAACTAGGAAACGAAGG-3'
88761_RACE_1_F	5'-GAAACCTAGCCAACTTACTGAAGGAGCC-3'
88761_RACE_2_F	5'-GGAACCCAAGACTGGCCAACGGTTG-3'
88761_GSP_R	5'-GCTAAGTAAGAAAGAAAGTAATTCACTGAGGTAAGTCG-3'
88761_RACE_1_R	5'-GGAACTGATACATTGTTTATTAGAAGAAATGGGG-3'
88761_RACE_2_R	5'-CTCTTTGACTAGGAGTTGCAACGATAAGTTCAAC -3'

Figure S1: Developmental stages of *Oxytricha*.
Various time points refer to the time passed since the mixing of compatible mating types to initiate conjugation. Cultures are not synchronized and time points are approximations based on (38).

Figure S2: qPCR validation of circular DNA enrichment and linear DNA depletion.
1ng of DNA before (-exo) and after (+exo) exonuclease treatment was used as input. Fold change represents relative amount of target in +exo sample compared to –exo sample and was calculated using $2^{(-\Delta Ct)}$ where $\Delta Ct = Ct_{+exo} - Ct_{-exo}$. pUC19 and TBE assays were done as technical triplicates, whereas the mitochondria levels were measured as technical duplicates. pUC19, a bacterial circular plasmid, was used to verify the enrichment of circular DNA upon exonuclease treatment. The mitochondrial *Oxytricha* genome is linear and was targeted to show the depletion of linear DNA upon exonuclease treatment.

Figure S3: Characteristics of high confidence rearrangement-specific eccDNA from nonrepetitive MIC-limited loci. A) MIC contigs are binned according to how many annotated high confidence eccDNA they contain. The contigs that do not have any eccDNA annotations are not shown. **B)** Length distribution of high confidence eccDNA. Bin sizes are 25bp and 250bp respectively for the two bar graphs. **C)** Pie chart represents the distribution of the location of 933 inferred cut sites for high confidence eccDNA in MDS-rich regions. **D)** Sequence similarities flanking eccDNA that have direct repeats >=1bp are shown as a pie chart. The repeat sequence and the number of eccDNA that have the particular direct repeat is shown. The pie chart only shows the distribution of direct repeat sequences that are present in at least 6 high confidence eccDNA. The table shows the longest and more complex direct repeats that rarely flank high confidence eccDNA.

Figure S4: Terminal transferase treatment of genomic DNA coupled to PCR to map 3' DNA breaks at MDS boundaries. Poly(G) tailed genomic DNA was PCR amplified to detect free 3' DNA ends for four MDSs: MDS 3 and 4 in MIC 67570, MDS 3 in MIC 88761 and MDS 13 in MIC 72447. Genomic DNA from cells during mid-rearrangement (Mid-R) and mixed parental cells JRB310 and JRB510 (P) as negative control was used. Control terminal transferase reactions lacking terminal transferase are represented by Tdt-.

Supplemental Figure 1

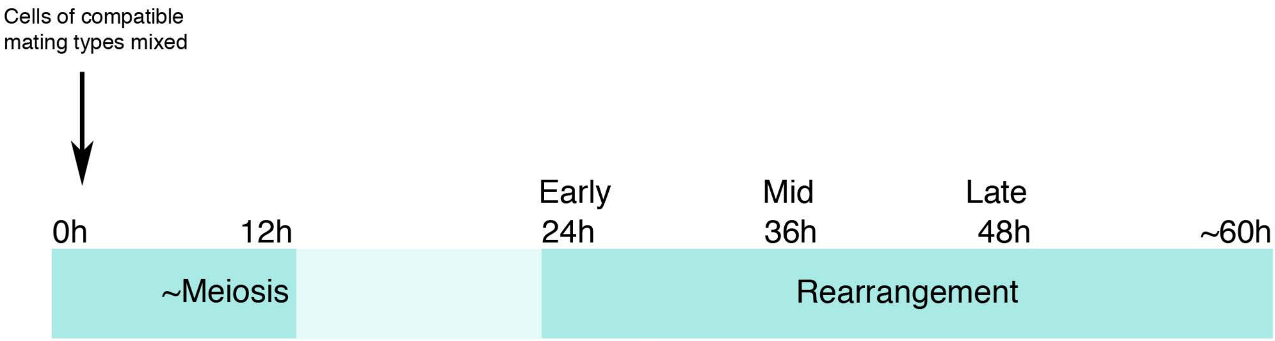

Supplemental figure 2

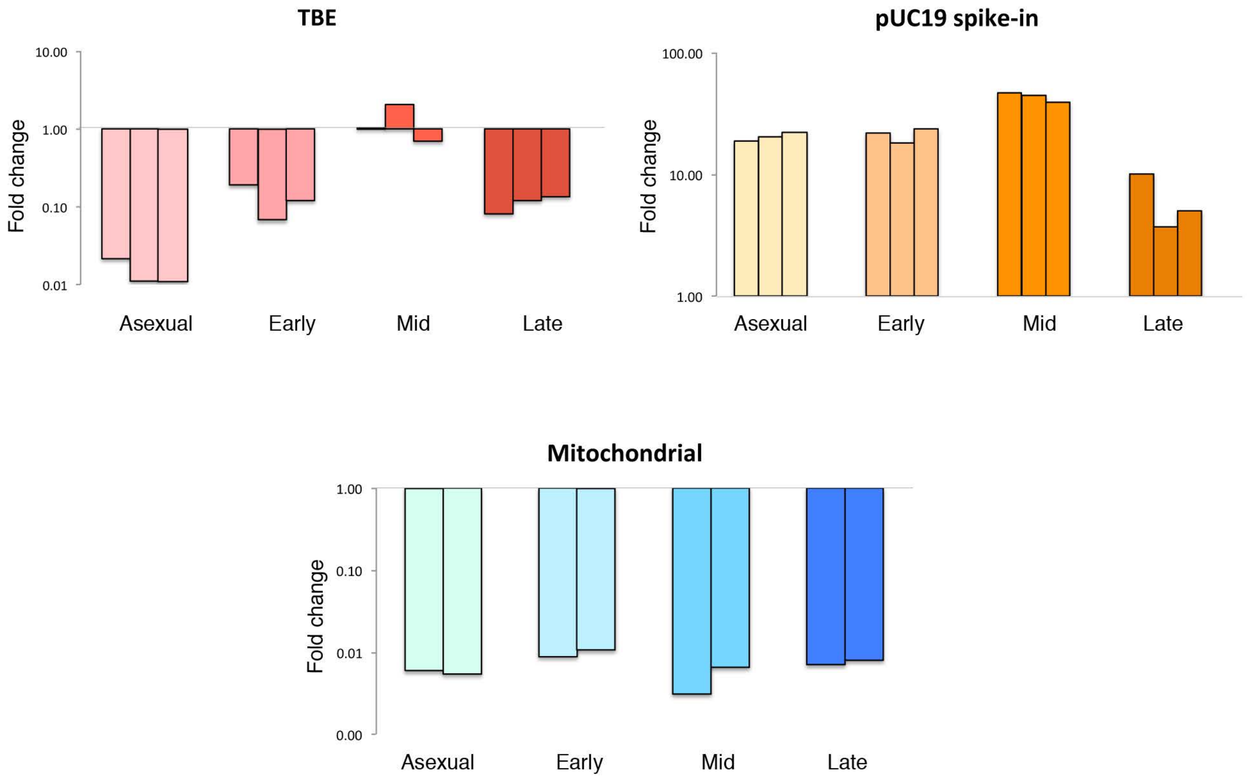

Supplemental figure 3

A

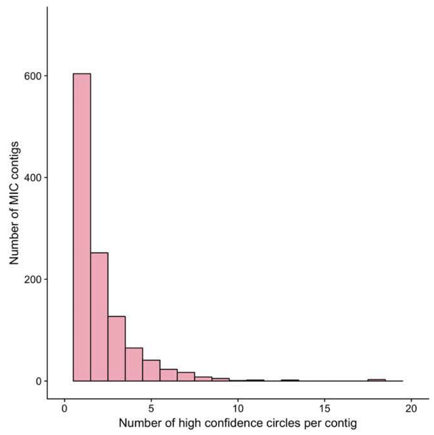

B

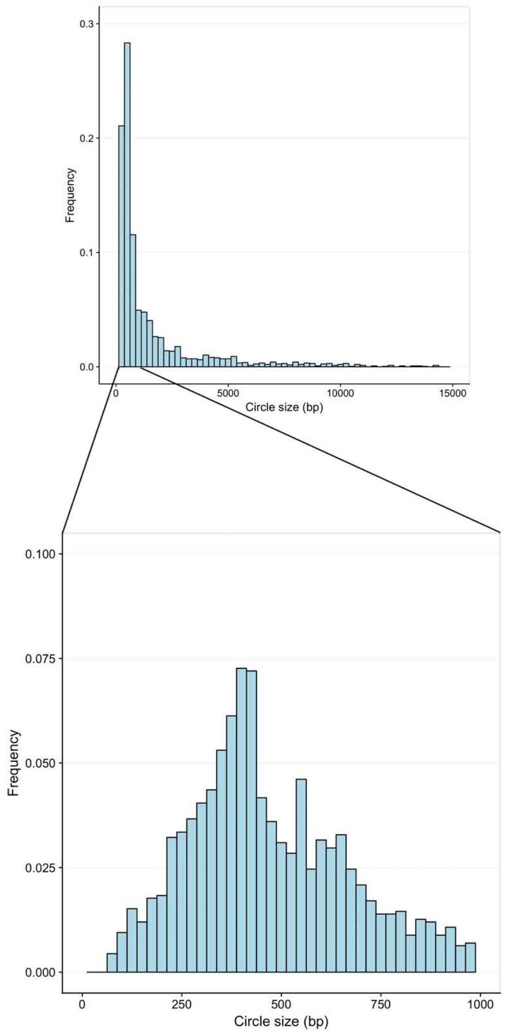

D

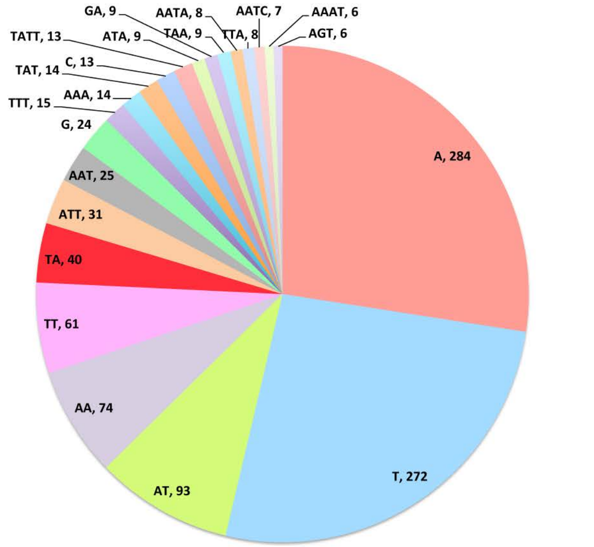

MIC contig	Circle start	Circle end	Repeat length	Repeat sequence
OXYTRI_MIC_37843	12331	12953	18	AATCTATCTCTATGCATT
OXYTRI_MIC_89621	41016	41707	16	TATCATGAAAAATCAT
OXYTRI_MIC_67551	104696	105382	15	AAATTTAATGTTATG
OXYTRI_MIC_85516	2433	2845	15	AGAAATTTCGGATTA
OXYTRI_MIC_77501	8764	9214	14	TTATAAATATAAAT
OXYTRI_MIC_87226	44282	44483	14	TGAAATGTTTAGTT
OXYTRI_MIC_87425	24058	24651	14	AATATTTTTAATTA
OXYTRI_MIC_68430	22256	22473	13	TATTAGTGTTCTT
OXYTRI_MIC_70325	30020	40648	13	AACAAAATAAAAT
OXYTRI_MIC_70604	9518	11465	13	ATAAACTCAACAA
OXYTRI_MIC_69002	6407	8023	12	TCCAGATTGGTG
OXYTRI_MIC_70746	37483	37770	12	TAAATAGCTGAT
OXYTRI_MIC_67635	40540	53805	11	ATTAATAAAAA
OXYTRI_MIC_68188	24540	24781	11	TTAATGCAGAT
OXYTRI_MIC_76142	11614	12053	11	TAATTTACTTG
OXYTRI_MIC_87739	38601	39237	11	AATGTGAACCC
OXYTRI_MIC_89929	19881	20294	11	TATTATTAATA
OXYTRI_MIC_67545	33559	33753	10	CCTTATTAAT
OXYTRI_MIC_67913	106374	106710	10	AGTTAGTTTT
OXYTRI_MIC_73579	15326	15860	10	TTTAATATAA

C

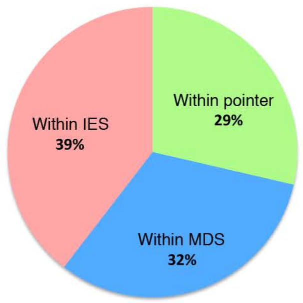

Supplemental figure 4

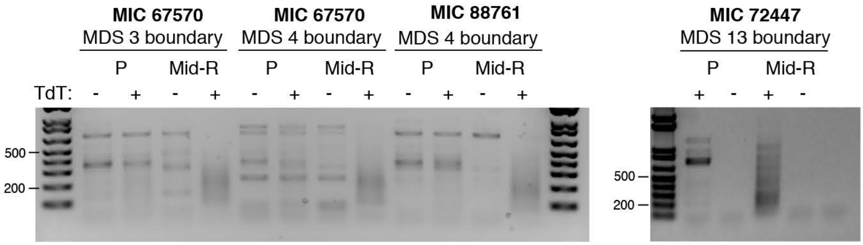

Supplement: gkz725_Supplemental_File [file gkz725_supplemental_file.pdf]
